# Neutralization of pertussis toxin by a single antibody prevents clinical pertussis in neonatal baboons

**DOI:** 10.1126/sciadv.aay9258

**Published:** 2020-02-05

**Authors:** Annalee W. Nguyen, Andrea M. DiVenere, James F. Papin, Sheila Connelly, Michael Kaleko, Jennifer A. Maynard

**Affiliations:** 1 Department of Chemical Engineering, University of Texas at Austin, Austin, TX 78712, USA.; 2 Division of Comparative Medicine, Department of Pathology, University of Oklahoma Health Sciences Center, Oklahoma City, OK 73104, USA.; 3 Synthetic Biologics, 9605 Medical Center Dr., Suite 270, Rockville, MD 20850, USA.

## Abstract

Pertussis continues to cause considerable infant mortality world-wide, which could be addressed in part by passive immunization strategies. Antibody hu1B7 is a candidate therapeutic that potently neutralizes pertussis toxin in vitro, prevents leukocytosis in mice and treats established disease in weanling baboons as part of an antibody cocktail. Here, we evaluated the potential for hu1B7 and an extended half-life hu1B7 variant to prevent death, leukocytosis and other clinical symptoms in a newborn baboon model that mimics many aspects of human disease. We administered a single antibody dose to newborn baboons five weeks prior to experimental infection. While all animals were heavily colonized with *Bordetella pertussis*, prophylaxed animals showed significantly greater survival (*P* < 0.005), delayed and suppressed leukocytosis (*P* < 0.01) and enhanced clinical outcomes, including coughing (*P* < 0.01), as compared to controls. Together, this work demonstrates that a single neutralizing anti-PTx antibody is sufficient to prevent clinical pertussis symptoms.

## Teaser

An antibody prevents the symptoms of whooping cough in newborn baboons when administered 5 weeks before infection.

## INTRODUCTION

Despite aggressive vaccination programs, pertussis remains a serious respiratory illness. Globally, this bacterial infection is responsible for ~1% of mortality in children under 5 years of age (*1*) or ~160,000 under-five deaths annually (*2*), with most cases occurring in Southeast Asia and Africa. Pertussis is of greatest concern for unimmunized infants, as they experience the highest mortality and most severe symptoms, including pneumonia and pulmonary hypertension due to severe leukocytosis with lymphocytosis (*3*). Despite general improvements in childhood morbidity and mortality due to pertussis in recent decades, rates for the youngest and most vulnerable infants have changed very little in low- and middle-income countries (*4*). In the United States, ~10% of reported cases require hospitalization (*5*), incurring considerable costs (*6*), and long-term morbidity.

*Bordetella pertussis* produces a wide array of toxins and adhesins, yet several lines of evidence point to the pertussis toxin (PTx) as a critical virulence factor (*7*). PTx is a classic AB_5_ toxin that binds sialylated glycoproteins on the target cell surface and then undergoes endocytosis and retrograde transport to the endoplasmic reticulum. There, the enzymatically active S1 subunit escapes into the cytosol to adenosine 5′-diphosphate–ribosylate, the α subunit of G_i/o_ proteins, thereby interfering with natural signal processing pathways (*8*). While circulating strains of *B. pertussis* have largely lost expression of the vaccine antigen pertactin, these same strains harbor the *PTxP3* promoter polymorphism, which increases PTx production (*9*). Conversely, strains lacking PTx are rarely observed and usually only in the context of immunocompromised individuals or mixed infections, in which PTx is supplied in trans by a strain expressing PTx (*10*). Clinically, PTx is directly responsible for the leukocytosis that marks severe cases (*11*).

Accordingly, PTx is a primary component of all licensed acellular vaccines, and high anti-PTx antibody levels are generally considered to correlate with protection (*12*), although no specific protective titer has been identified (*13*). A high-dose, PTx-only vaccine has been used in Denmark since 1997 and appears equally efficacious as the multicomponent acellular vaccines used elsewhere (*14*). Passive immunization with anti-PTx serum was evaluated as a potential therapeutic modality for neonatal pertussis and showed promise, but this product is no longer produced (*15*). Currently, maternal vaccination is the leading strategy to protect newborns from pertussis before initiating standard vaccination schedules at 6 to 8 weeks of age. The goal is to induce high levels of maternal anti-pertussis antibodies for transfer to the fetus in utero that then confer protection after birth (*16*).

While maternal vaccination is attractive for many reasons, it is unlikely to capture all eligible mothers during the appropriate gestational period. Moreover, the resulting infant anti-pertussis titers can be fairly modest at birth and decay rapidly (*17*). Prophylaxis with a single neonatal dose of a potently neutralizing monoclonal antibody provides a complementary approach to protect infants whose mothers were not immunized during pregnancy. This approach may be valuable for infants at high risk of severe pertussis, such as those born prematurely or with low birth weight in geographical areas of high disease burden (*11*). Antibody prophylaxis would result in very high neutralizing anti-PTx titers and, when engineered for extended serum half-life, could provide protection during the first 4 months of life or longer. Moreover, passive immunization of neonates presents a best-case scenario for maternal immunization, in which the concentrations, binding specificities, and neutralizing activities of the anti-pertussis antibodies are tightly controlled and the impact of changing antibody titers on clinical metrics can be directly observed.

We previously described the anti-PTx antibody hu1B7 and determined that it protects against toxin activities by altering intracellular transport steps required for PTx to access its target (*18*). When administered to mice, hu1B7 prevented leukocytosis in intoxication (*19*) and infection models (*20*) and reduced *B. pertussis* lung colonization levels ~30-fold (*20*). As part of an antibody cocktail composed of two PTx-neutralizing antibodies, hu1B7 treated leukocytosis in weanling baboons with established disease (*20*). Here, we hypothesized that hu1B7 administration at birth would provide protection from severe pertussis and death. An extended half-life version of hu1B7, hu1B7-YTE, was generated, and the prophylactic potential of both hu1B7 variants to protect infants during their first few months was evaluated using a recently described neonatal baboon model of disease that mimics many aspects of human disease (*21*).

## RESULTS

The humanized hu1B7 antibody and its murine predecessor m1B7 are protective in many in vitro and in vivo assays of PTx activity and *B. pertussis* infection (*20*, *22*). With information regarding the cellular mechanisms by which hu1B7 neutralizes PTx as well as promising prior mouse and baboon protection data (*20*), we wondered whether hu1B7 could independently protect baboon neonates in a prophylactic setting that closely mimics maternal immunization. Two antibody lots were produced and purified as human immunoglobulin G1 (IgG1) isotypes: hu1B7 and an extended half-life variant, hu1B7-YTE, containing three amino acid changes in the CH_2_ domain (M252Y, S254T, and T256E). These YTE changes were previously shown to extend antibody half-lives ~4-fold as compared to the unmodified antibody in monkeys and humans by virtue of increased antibody affinity for the neonatal Fc receptor (FcRn) at endosomal pH values and the resulting enhanced antibody recycling of internalized antibodies to the serum (*23*–*25*).

Our prior work suggested that hu1B7 does not rely heavily on Fc functions to neutralize PTx activities (*18*). To formally evaluate this hypothesis and understand the potential for Fc changes such as YTE to affect protection, we generated an aglycosylated hu1B7 variant and compared it to hu1B7 in a mouse *B. pertussis* challenge model. hu1B7 was deglycosylated by virtue of an asparagine-to-alanine substitution at position 297 (hu1B7-N297A), which greatly reduces binding to complement and Fcγ receptors while not affecting FcRn binding or antibody half-life. Since the YTE changes paradoxically accelerate antibody clearance in mice due to increased affinity for the mouse FcRn homolog at neutral pH (*23*), hu1B7-YTE was not included in this experiment. All antibody preparations had similar purity, as judged by SDS–polyacrylamide gel electrophoresis, and bound PTx with similar affinities, as measured by enzyme-linked immunosorbent assay (ELISA) (fig. S1).

**Fig. 1:**
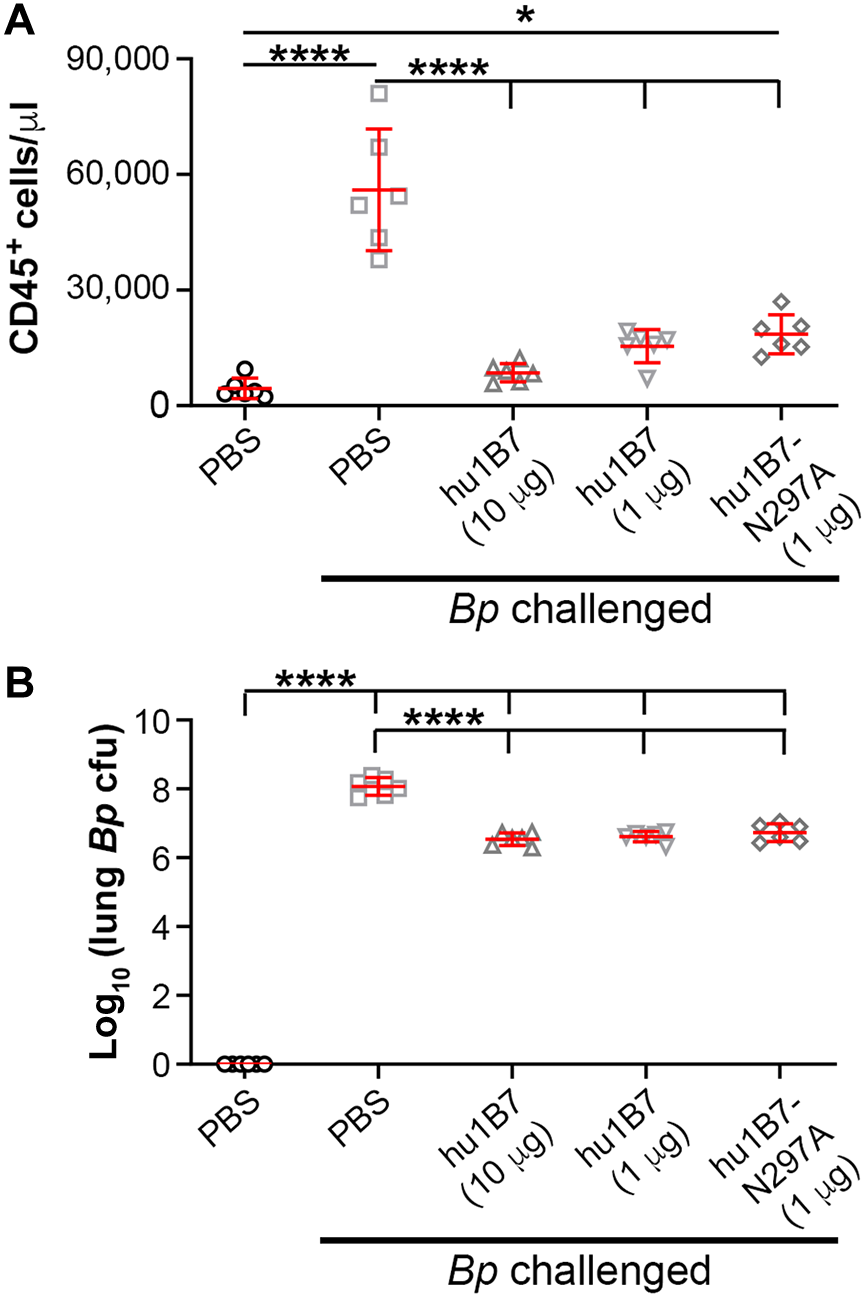
Hu1B7 and aglycosylated hu1B7-N297A antibodies confer similar protection against *B. pertussis* challenge in mice. Four-week-old Balb/c mice were administered antibody or PBS intraperitoneally in a 200-μl volume 2 hours before infection with 10^7^ CFU *B. pertussis* (*Bp*) strain D420. Infection severity was assessed after 7 days by (**A**) CD45^+^ leukocytosis (WBC) and (**B**) *B. pertussis* lung colonization (no colonies were recovered for PBS-treated, uninfected mice). Significance was determined by two-way analysis of variance (ANOVA) with Tukey post hoc test; *P < 0.05; ****P < 0.0001.

To compare protection conferred by hu1B7 and hu1B7-N297A, we administered a minimally protective antibody dose (1 μg; fig. S2) to Balb/c mice 2 hours before intranasal challenge with 10^7^ colony-forming units (CFU) of *B. pertussis* strain D420. For comparison, a high dose of hu1B7 (10 μg) or phosphate-buffered saline (PBS) mock treatment were administered as controls. After 7 days, mice were evaluated for CD45^+^ leukocytosis and lung *B. pertussis* colonization levels. PBS-treated animals had high white blood cell (WBC) counts (~50,000/μl), which were significantly suppressed in all antibody-treated animals (5000 to 10,000/μl; *P* < 0.0001), with only hu1B7-N297A having a slightly but significantly elevated WBC as compared to uninfected animals (*P* < 0.05; Fig. 1A). Lung *B. pertussis* colonization levels were also suppressed ~30-fold with antibody treatment (Fig. 1B), similar to our previous observations (*20*). Collectively, these data indicate that Fc functions play a minor role in hu1B7-mediated protection and suggest that hu1B7-YTE retains a similar protective capacity as hu1B7. Even at low hu1B7 dosages of 0.25 μg/15 g mouse, equivalent to 0.017 mg/kg, the WBC was only modestly elevated and lung *B. pertussis* colonization suppressed, underscoring the high potency of this antibody (fig. S2).

Accordingly, we proceeded to assess protection conferred by hu1B7 and hu1B7-YTE in a neonatal baboon model of pertussis infection. To facilitate comparisons, we designed our study to closely mimic a prior study in which pregnant baboons were immunized and their neonates subsequently infected (*21*). Neonatal baboons with no baseline anti-filamentous hemagglutinin (Fha) or anti-PTx serology, indicating they had not been previously exposed to *Bordetella* species (*26*), were recruited into this study and administered antibody (40 mg/kg) or nothing intravenously on day 2 of life, after which they were hand-raised and bottle-fed. Antibody pharmacokinetics were monitored for approximately 5 weeks, at which time the animals were challenged with *B. pertussis* strain D420 and followed for an additional 6 weeks. Seven control, eight hu1B7, and seven hu1B7-YTE animals of both genders were recruited, with no significant differences between groups in terms of age, body weight, or baseline anti-Fha titer (Table 1).

**Table 1:** Summary of baboon enrollment and infection data. N/A, not applicable.

**Treatment** **group**	**Age at** **infection** **(days)**	**Weight at** **infection** **(kg)**	**Anti-Fha** **titer at** **infection** **(IU/ml)**	**Antibody** **β–half-life** **(days)**	**Anti-PTx** **titer at** **infection** (μg/ml)*	***B. pertussis*** **CFU (×10^7^/ml,** **day 3/4)**	**Anti-Fha** **titer, day** **20/21** **(IU/ml)**	**Max WBC** (*1000/ μl)*	Survival*
**Control (C)**	32.3 ± 3.9	1.2 ± 0.1	1.8 ± 1.5	N/A	0.2 ± 0.3	3.3 ± 3.3	66 ± 98 (*n* = 3)	40.8 ± 14.9	3/7
**hu1B7^†^ (T)**	34.3 ± 3.9	1.4 ± 0.2	1.9 ± 1.6	12 ± 2	41 ± 12	1.49 ± 0.69	34 ± 29	14.8 ± 4.7	7/7
**hu1B7-YTE (Y)**	32.9 ± 2.1	1.3 ± 0.2	1.4 ± 2.4	20 ± 5	94 ± 32	15.6 ± 31	30 ± 36	16.5 ± 9.9	7/7

*Only the following categories showed significant differences between treatment groups: antibody titer on day of infection (hu1B7 versus hu1B7-YTE, *P* < 0.01), survival (hu1B7 or hu1B7-YTE versus controls, *P* < 0.005), and maximum WBC (hu1B7 or hu1B7-YTE versus controls, *P* < 0.01).

†Animal T2 had no detectable hu1B7 titer at the time of infection and was excluded from pharmacokinetic and efficacy analyses on the basis of the Grubb’s test for outliers with α = 0.05.

**Fig. 2:**
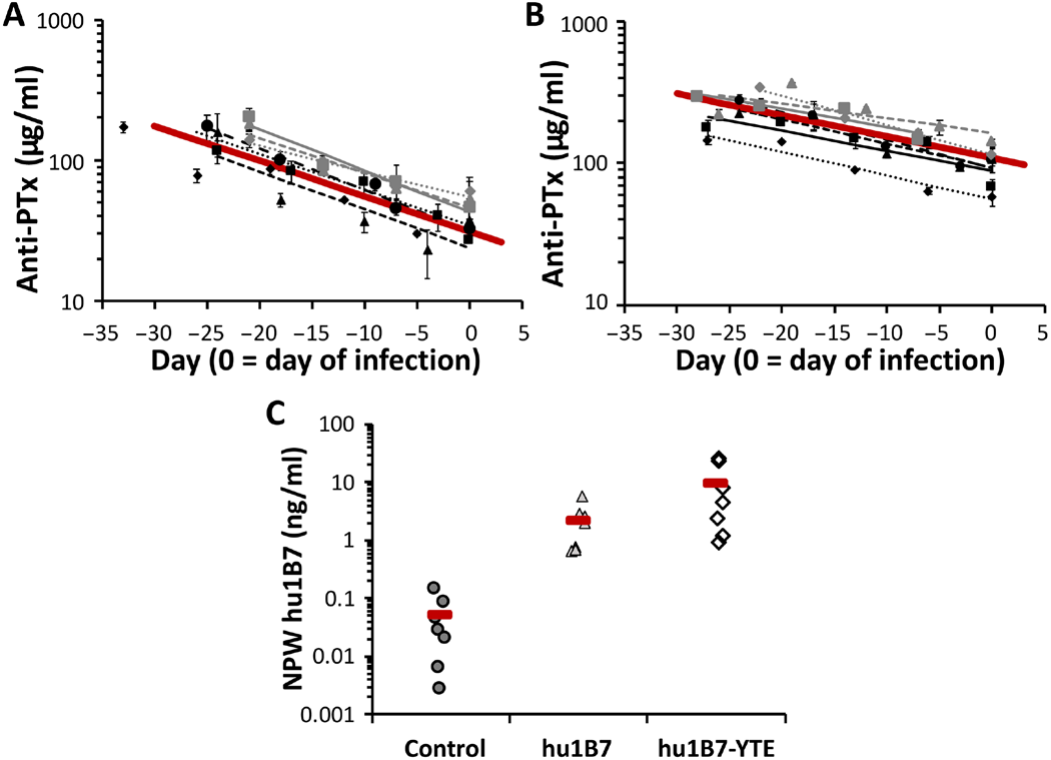
Extended half-life antibody hu1B7-YTE persists longer than hu1B7 in neonatal baboons. Serum antibody concentrations were determined by PTx ELISA and fit to determine the β-decay rate and serum half-life of (**A**) hu1B7 and (**B**) hu1B7-YTE before infection. Data for each treated animal are shown with a unique symbol and line fit with the average decay for each antibody shown in a bold red line. The two datasets are significantly different (*P* < 0.01) on the basis of the two-tailed *t* test assuming unequal variances with α = 0.05. (**C**) Hu1B7 was detected in the nasopharyngeal wash (NPW) samples of animals 2 to 4 days after infection, which corresponds to 29 to 42 days after antibody infusion. Each group mean is shown as a red bar (*n* = 7 per group). Data for all animals are shown except for hu1B7-treated animal T2, which was excluded from this analysis on the basis of the Grubb’s test for outliers with α = 0.05.

Since there was a significant delay between antibody administration and pertussis challenge, it was important to carefully track the antibody pharmacokinetics and determine the serum hu1B7 concentration at the time of infection. Accordingly, serum was collected and the titer measured weekly before infection and biweekly thereafter. Preinfection titers fitting a single exponential decay rate were used to calculate the observed antibody half-life for each animal and then averaged across all animals in each treatment group. The half-life of hu1B7 was measured as 12 ± 2 days, while the half-life of hu1B7-YTE was significantly extended to 20 ± 5 days (*P* < 0.01; Fig. 2, A and B). This resulted in serum titers of 41 ± 12 μg/ml for animals receiving hu1B7 and 94 ± 32 μg/ml for those receiving hu1B7-YTE at the time of infection. One hu1B7-treated animal (T2), cleared the antibody very rapidly [half-life (*t*_1/2_) = 3.0 days; 4.5 SDs below the mean for this treatment group], such that the hu1B7 titer had returned to the preinfusion baseline level at the time of infection. Since this study was designed to evaluate the impact of hu1B7 on clinical outcomes after infection and animal T2 met objective statistical metrics for exclusion as an outlier using the Grubb’s test, this animal only was excluded from pharmacokinetic and efficacy analyses (detailed data for all animals shown in table S1 and figs. S3 and S4).

*B. pertussis* is a respiratory infection and while PTx exerts systemic effects (*27*), it is expected that anti-PTx antibodies are also needed at the mucosal surface to protect neutrophils mediating bacterial clearance. Thus, anti-PTx antibodies may indirectly support bacterial clearance, as reduced *B. pertussis* lung colonization was observed in prior mouse and adolescent baboon studies with hu1B7 (*20*). Both hu1B7 and hu1B7-YTE were detected in nasopharyngeal wash samples 2 to 4 days after infection (29 to 42 days after antibody infusion), albeit at substantially lower levels than in sera (Fig. 2C).

**Fig. 3:**
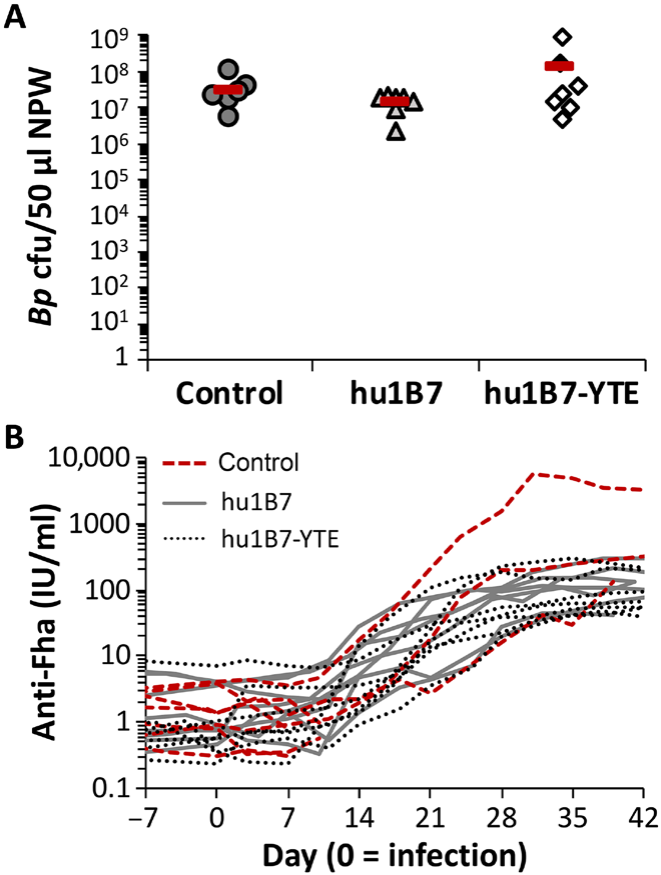
Anti-PTx prophylaxis did not affect initial colonization with *B. pertussis* or antibody response to Fha. (**A**) Nasopharyngeal washes collected from each baboon 3 to 4 days after infection were serially diluted and plated on selective media to quantify the *B. pertussis* CFU recovered (group means shown as a bar; *n* = 7 per group). No significant differences between treatment groups were observed (ANOVA; α = 0.05, *P* = 0.3). (**B**) Serum anti-Fha titers were determined by ELISA twice weekly throughout the experiment. The baboons had varied, but not significantly different, primary responses to Fha by treatment group; compared on day 27/28 by ANOVA (α = 0.05, *P* = 0.08; note that only three of seven control animals survived to this date).

Approximately 5 weeks after antibody administration, all animals were experimentally infected with 10^8^ CFU of *B. pertussis* strain D420. All animals were heavily colonized by *B. pertussis* when first assessed 3 to 4 days after experimental infection, with no significant differences between groups observed, indicative of a successful infection (Fig. 3A). For surviving animals, colonization remained high until declining 3 to 4 weeks after infection (fig. S3). Since only three of seven control animals survived to provide a full dataset for comparison, we are unable to determine whether antibody presence accelerated bacterial clearance. Anti-Fha antibody titers increased ~10-fold in all animals ~12 to 21 days after infection, consistent with a robust primary immune response (Fig. 3B).

**Fig. 4:**
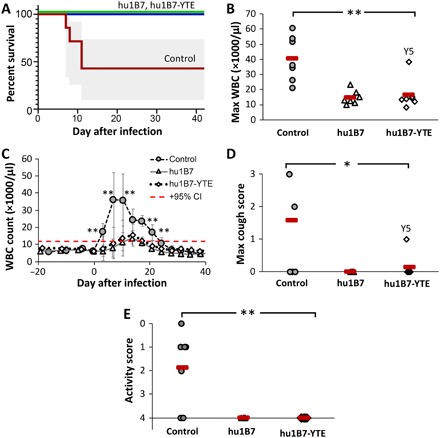
Hu1B7 and hu1B7-YTE administration reduces pertussis mortality, leukocytosis, and clinical symptoms in neonatal baboons. (**A**) All antibody-treated animals survived infection, while four of seven controls required euthanasia [95% confidence intervals (CI) in gray; *P* = 0.0023 for each treated group versus controls; log-rank test]. (**B**) The maximum WBC count was significantly reduced in antibody-treated baboons relative to controls (**P < 0.01; ANOVA, α = 0.05, *P* = 0.0003; post hoc comparison with Tukey post hoc test). Hu1B7-treated animal T2 was excluded from this analysis on the basis of the Grubb’s test for outliers with α = 0.05. (**C**) Leukocytosis was delayed and suppressed for antibody-treated animals (group averages ± SD shown; data for individual animals in fig. S2). The control group WBC was significantly higher than treated groups on days indicated (**P < 0.01), and the onset of leukocytosis was delayed from day 3 for control animals to days 10 to 14 for treated animals (*P* < 0.01). The dashed red line indicates the upper 95% CI for preinfection WBC and defines WBC elevation. (**D**) Clinical signs showed reduced maximum cough count (0, none; 1, occasional; 2, frequent; and 3, severe and frequent) with antibody treatment (*P < 0.05 control versus either treated group; ANOVA, α = 0.05, *P* = 0.005; post hoc comparison with Tukey post hoc test) and (**E**) improved activity scores (0, immobile and requires euthanasia; 1, very little activity; 2, reduced movement and no jumping/climbing; 3, movement but reduced jumping/climbing; and 4, normal activity) for treated versus control animals (**P < 0.01; ANOVA, α = 0.05, *P* = 0.005; post hoc comparison with Tukey post hoc test).

In terms of efficacy, all control animals developed significant leukocytosis and four met stringent euthanasia criteria. By contrast, all antibody-treated animals survived (*P* < 0.005; Fig. 4A). Using the maximum observed leukocytosis as a metric, WBC counts for all treated animals remained significantly lower than controls (*P* < 0.01; Fig. 4B). Inspection of the leukocytosis time course shows a rapid WBC increase after infection for control animals, which is delayed and suppressed in antibody-treated animals. Control animals exhibited an average onset of leukocytosis (defined as the postinfection day that the WBC rose above the 95% confidence interval for preinfection WBC levels) on day 3, while antibody-treated groups reached this point on days 10 and 14 after infection (*P* < 0.01). Comparing individual days after infection, leukocytosis was significantly lower in antibody-treated animals on all days 3 to 21 after infection (*P* < 0.01), except for day 14, and the WBC elevation lasted for a shorter period (3 to 7 days for treated animals versus 21 days for controls, *P* < 0.05; Fig. 4C and fig. S4). Coughing and lethargy were also exacerbated in control animals as compared to antibody-treated animals (*P* < 0.05), which generally appeared clinically normal (Fig. 4, D and E).

Two antibody-treated animals merit special attention: infants born in different years to the same mother who received hu1B7 (animal T2) or hu1B7-YTE (animal Y5). Both animals exhibited unusually rapid antibody clearance kinetics: T2 cleared the hu1B7 antibody before infection, while Y5 cleared it 10 days after infection (fig. S5). This appears due to the presence of antidrug antibodies recognizing hu1B7 and hu1B7-YTE, as their concentration on the day of infection correlates tightly with the antibody concentration 7 days after infection (−0.95 Pearson correlation coefficient; fig. S6). While several hu1B7-treated animals developed antidrug antibodies, they were only observed in one hu1B7-YTE–treated animal (Y5), suggesting that this is not an intrinsic characteristic of the antibody (fig. S5). Similar phenomena have been observed with other human antibodies after administration to nonhuman primates (*28*).

**Fig. 5:**
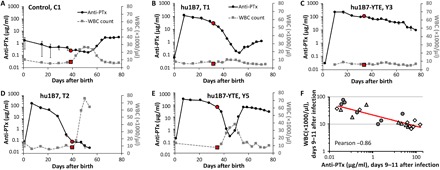
Anti-PTx antibody concentrations correlate inversely with leukocytosis. (**A**) Control animals exhibited rapid onset of leukocytosis after infection. For control animals such as C1 that were ultimately able to control infection and did not require euthanasia, leukocytosis diminished rapidly upon appearance of an endogenous anti-PTx response. (**B**) Most antibody-treated animals exhibited typical antibody clearance kinetics with an early α-decay, followed by a slower β-decay phase, as seen in animal T1. Endogenous anti-PTx antibodies appeared ~12 days after infection but were not always distinguishable from the remaining hu1B7 background. (**C**) This is apparent in animal Y3, which cleared hu1B7-YTE more slowly than hu1B7. Other baboons had evidence of α, β, and additionally γ-decay kinetics, which correlate closely with the WBC rise and appearance of antidrug antibodies (fig. S4). This was especially pronounced and early in animals (**D**) T2 and (**E**) Y5, which shared the same mother. (**F**) Anti-PTx titers correlate most strongly with WBC counts when both are measured on days 9 to 11 after infection, just after disease is most severe, with control animals shown in circles, hu1B7-treated animals in triangles, and hu1B7-YTE–treated animals in diamonds. The day of infection is indicated by a red marker in both the anti-PTx and WBC data plots. Comparisons for all animals are shown in fig. S3.

These animals allow a careful comparison of the relationship between anti-PTx titers and leukocytosis. As our reagents are unable to differentiate between human and baboon IgG, we used the timing after infection to discriminate between hu1B7 antibody titers and the endogenous polyclonal baboon responses to PTx, which appear ~12 days after infection. For surviving control animal C1, a rapid WBC increase was observed after infection, which decreased with the advent of endogenous anti-PTx titers on day 21 (Fig. 5A). For the fully protected hu1B7 recipient T1, hu1B7 titers were high at the time of infection, decreased to a nadir on day 21, at which point baboon anti-PTx responses rose. For this animal, the WBC remained low (<10,000/μl) for the entire experiment (Fig. 5B). The hu1B7-YTE recipient Y3 showed a similar low WBC, with slower antibody clearance kinetics characteristic of hu1B7-YTE that obscure the appearance of baboon responses (Fig. 5C). As noted above, animal T2 cleared the hu1B7 antibody before infection and was not protected; this animal required euthanasia (Fig. 5D). Animal Y5 also cleared hu1B7-YTE relatively rapidly after infection: This animal was partially protected (Fig. 5E) and was the only antibody-treated animal to cough (Fig. 4D). Y5’s WBC rose only during the period of low total anti-PTx titers between days 7 and 14: after hu1B7-YTE was cleared and before the endogenous anti-PTx titers rose. Similarly, animal T4 exhibited slightly lower hu1B7 concentrations and more rapid clearance than other hu1B7-treated animals that corresponded with a rapid onset of leukocytosis (fig. S4). Comparisons for all animals are shown in fig. S4.

Collectively, these data suggest that PTx-neutralizing antibodies are required at all times when colonized by *B. pertussis* to fully protect against leukocytosis and severe pertussis. Furthermore, they suggest that a minimal hu1B7 concentration required for protection could be defined and, more broadly, that neutralizing anti-PTx titers could be used as a serological correlate of protection from leukocytosis. This point is supported by correlational analyses of hu1B7 titers versus WBC across all treated animals, which are high (Pearson ≤ −0.79) when comparing the anti-PTx titer on days 0, 3/4, 6/7, and 9/10/11 with all combinations of WBC on days 3/4, 6/7, and 9/10/11, and the maximum WBC count. This correlation is strongest when comparing days 9 to 11 anti-PTx titers, typically days with the lowest hu1B7 titer before endogenous baboon responses appear, with the WBC on these same days (−0.86 Pearson; Fig. 5F). Since disease in this model is most severe on day 7 (*29*), the correlation implies a need for neutralizing anti-PTx antibodies when the disease is most serious. The weakest correlation was the anti-PTx titer on the day of infection with that animal’s maximum WBC count obtained.

Last, we next wanted to explore whether hu1B7 presence would interfere with subsequent acellular pertussis vaccination, using a formulation containing equal amounts of chemically inactivated PTx and Fha. We administered mice a modified hu1B7 with murine IgG2a constant domains (reverse chimera or r-ch1B7; fig. S1) to retain normal pharmacokinetics and then vaccinated the mice with the human acellular pertussis vaccine 6 hours later and boosted 4 weeks later. Mouse anti-Fha titers increased similarly for all immunized mice at all time points (fig. S7A). Unimmunized mice revealed r-ch1B7 titers to be high at the time of primary immunization, low at 4 weeks, and undetectable by 6 weeks after administration. Immunized mice showed similar high anti-PTx titers at 4 and 6 weeks, regardless of r-ch1B7 treatment (fig. S7B). The whole-cell vaccine was not included in this experiment because it was not included in this experiment because it contains only traces of PTx and is less likely to be affected by the presence of anti-PTx antibodies. Overall, these data suggest that the presence of an antibody binding a single PTx epitope may have minimal impact on total anti-PTx titers after acellular vaccination.

## DISCUSSION

While antibody therapeutics are primarily targeted toward treatment of cancer and inflammatory diseases, there is a growing interest in the use of antibodies to treat infectious diseases, with four antibodies now approved for infectious indications (*30*). Antibodies are appealing for their abilities to retain activity in the face of antibiotic resistance and to specifically target one species without harming the remaining microbiome. Moreover, antibody engineering efforts to enhance efficacy and serum half-life promise to reduce required dosages, while improvements in antibody manufacturing have increased yields, making antibodies an increasingly economical option. For these reasons, antibody prophylaxis is being considered to prevent diseases for which no vaccine is yet licensed or that predominately affect high-risk populations, such as neonates (*31*, *32*). Monoclonal antibody prophylaxis has been the leading strategy to protect infants from respiratory syncytial virus (RSV) since 1998 (*30*).

Here, we evaluated the potential for this clinical strategy to prevent neonatal pertussis, using a single PTx-neutralizing human IgG1 antibody in the recently described neonatal baboon model (*21*). This extends our prior report in which we treated adolescent baboons on day 3 after infection and observed an immediate halt in the WBC rise (*20*). In the previous study, we used a cocktail of two antibodies neutralizing PTx via complementary mechanisms but here used just one antibody to reduce manufacturing costs. In this study, we administered 40 mg/kg of the hu1B7 antibody, a variant engineered for extended half-life in humans called hu1B7-YTE or nothing to baboons on day 2 of life. We then followed the antibody pharmacokinetics for approximately 5 weeks before experimental infection with a recent clinical isolate of *B. pertussis*. While all animals were heavily and similarly colonized with *B. pertussis*, as expected, prophylaxed animals showed significantly greater survival (*P* < 0.005), suppressed leukocytosis (*P* < 0.01), and enhanced clinical outcomes (*P* < 0.05) as compared to controls (Fig. 4). Inspection of the antibody pharmacokinetics revealed a strong correlation between anti-PTx titers and leukocytosis (Fig. 5).

In baboons, these antibodies exhibited half-lives of 12 ± 2 and 20 ± 5 days for the hu1B7 and hu1B7-YTE variants, respectively. This is similar to a prior report that administered human IgG1 RSV-neutralizing antibody to cynomolgus macaques (30 mg/kg) and reported a ~4-fold improvement in half-life from ~6 days for the wild-type Fc to ~21 days for the YTE variant (*24*). These same anti-RSV antibodies exhibited a ~4-fold improved half-life in humans, from 19 to 34 days for the wild-type Fc to 70 to 100 days for the YTE version, depending on the dose administered (*25*, *33*). The shorter half-lives in nonhuman primates versus humans are presumed because of altered binding kinetics between the human antibody and nonhuman FcRn protein. In human infants, hu1B7 and hu1B7-YTE are expected to exhibit substantially longer half-lives and provide more durable prophylaxis than the baboon data reported here, similar to the half-lives observed for the anti-RSV antibodies. Extrapolating from the data shown here and the measured half-lives of YTE-containing antibodies in humans, antibody administration at birth could potentially provide >5 months of protection and may markedly reduce the cost of goods for implementation in the developing world.

The YTE changes used to extend half-life do negatively affect Fcγ receptor binding, specifically, binding to FcγRIIIa/CD16 involved in antibody dependent cytotoxicity (*28*, *34*). A second set of amino acid changes, M428L/N434S in the CH_3_ domain, have been shown to confer a similar half-life extension in nonhuman primates with less effect on Fcγ receptor binding but had not been evaluated in humans at the time this study began and was not selected for this reason (*28*). To determine whether the YTE changes would decrease efficacy of a hu1B7-YTE antibody, we first evaluated protection conferred by aglycosylated hu1B7-N297A in mice at a minimally protective dose. Human antibodies can mediate effector functions in mice, with compromised FcγRIIa binding, while aglycosylated antibodies lack binding to all mouse and human Fcγ receptors (*35*). Consistent with our prior report suggesting a minimal role for Fc functions in hu1B7-mediated protection (*18*), hu1B7-N297A provided nearly the same protection against leukocytosis as hu1B7 (Fig. 1). Similarly, the hu1B7-YTE variant showed comparable efficacy as hu1B7 in the baboon neonate, despite potential inefficiencies related to interactions between a human Fc interacting and baboon Fcγ receptors (Fig. 4).

PTx was selected as the target in this study, since it is directly responsible for leukocytosis, which is, in turn, predictive of clinical outcomes (*11*). In human infants, extreme leukocytosis leads to formation of leukocyte aggregates in lung arterioles and venules, which are thought to portend irreversible pulmonary hypertension and death (*3*). Clinical efforts to intervene in this sequence of events have included leukofiltration and exchange blood transfusion to reduce the WBC (*36*). Pharmacological strategies to interfere with downstream signaling events affected by PTx are also under investigation, such as repurposing of U.S. Food and Drug Administration–approved S1-P receptor agonists to sequester lymphocytes and reduce pulmonary inflammation (*37*). By contrast, the hu1B7 antibody blocks this cascade by preventing PTx from ever reaching its intracellular target.

The experimental design used here is very similar to that used in prior maternal immunization studies with the neonatal baboon model, allowing a comparison of these two clinical strategies. A key difference is that maternal immunization elicits a polyclonal antibody response binding numerous PTx epitopes, of which only a fraction are neutralizing, while hu1B7 prophylaxis provides a high concentration of only PTx-neutralizing antibodies. The prior studies enrolled neonatal baboons born to mothers vaccinated with diptheria-tetanus-acellular pertussis vaccine (DTaP) or a PTx-only vaccine during gestation, also infecting them with *B. pertussis* at about 5 weeks of age. Infants born to DTaP-vaccinated mothers had total anti-PTx titers of ~100 international units (IU)/ml at the time of infection (*21*), while those whose mothers were immunized with a PTx-only vaccine had lower initial titers ~10 IU/ml (*38*). These results are similar to ours in that only animals with high total anti-PTx titers exhibited enhanced survival, suppressed leukocytosis, and normal clinical signs. A ~3-fold increase in maximum leukocytosis was observed (*38*), which is similar to the transient ~2-fold increase observed here (Fig. 4 and fig. S3). In the two prior studies and this one, control animals coughed, while those receiving vaccine or having sufficient hu1B7 titers did not. Overall, antibody prophylaxis provided similar clinical benefits to neonatal baboons as maternal immunization and could provide a longer duration of protection in humans.

The results of the current study support maternal immunization programs striving for high and sustained neutralizing anti-PTx titers in infants. Data from individual animals (e.g., Y5) suggest that a titer >1 μg/ml hu1B7 is required for protection in this model and that this titer needs to be maintained until the infant’s immune system supplies a similar level of neutralizing anti-PTx antibodies. Human clinical data have demonstrated a correlation between the dose and detoxification method of PTx in a vaccine formulation and subsequent postvaccination anti-PTx titers, with the Danish high dose PTx only (*14*) and genetically inactivated PTx (PTg) (*39*) conferring the highest and most durable responses. Moreover, prior data from us and others demonstrated that PTg preserves the hu1B7 epitope and induces higher titers of hu1B7-like neutralizing antibodies than chemically detoxified PTx (*40*, *41*). Considering the complications of repeated dosing with Tdap (tetanus-diptheria-acellular pertussis) combination vaccines and the clinical benefits of hu1B7-like antibodies shown here, our results support use of a PTg-only maternal vaccine.

A related consideration in maternal vaccination strategies relevant for hu1B7 prophylaxis is the potential for residual antibodies to inhibit infant responses to the primary vaccination schedule. Specifically, there is some evidence to support reduced total anti-PTx titers in infants whose mothers were vaccinated during pregnancy, but it is unclear whether these affect efficacy (*42*). Antibody prophylaxis poses similar concerns. On one hand, these concerns are potentially diminished since hu1B7 binds a single epitope, whereas the polyclonal maternal response targets multiple epitopes. A polyclonal response could mask multiple epitopes and accelerate PTx clearance through formation of immune complexes. On the other hand, vaccination in the presence of hu1B7 could result in high overall anti-PTx titers but low levels of antibodies binding the highly neutralizing hu1B7 epitope. While this is possible, responses to other neutralizing epitopes not recognized by hu1B7, such as the receptor binding epitope on the B subunit, are less likely to be affected and are also protective in animal infection models (*20*, *22*). Our initial assessment suggests that the presence of 1B7 antibodies does not inhibit the total polyclonal response to PTx following acellular vaccination (fig. S7). Moreover, antibody-treated baboons developed endogenous responses to PTx in the presence of hu1B7 that fully suppressed WBC elevation after hu1B7 clearance (fig. S5).

While this study addressed many of the limitations from our prior study (*20*), others remain. For instance, prophylactic administration precludes the need for rapid identification of infected infants. However, the cost of an antibody therapeutic remains high relative to maternal vaccination, although this may be ameliorated by future in situ production strategies from administered nucleic acids (*43*). Furthermore, nonhuman primates are inherently highly variable: Animal T2 completely cleared hu1B7 before infection, resulting in behavior consistent with an untreated animal (and was excluded as an outlier), while Y5 cleared the antibody more rapidly than most animals but still retained sufficient hu1B7-YTE for partial protection. The antidrug antibody titers were very high in these two animals and presumably mediated hu1B7 clearance, possibly because of a shared major histocompatibility complex allele driving the antidrug responses. Since this study retained seven animals per treatment group, our overall results attained a high level of significance, despite this unexpected occurrence. Regardless, immunogenicity in nonhuman primates is poorly predictive of immunogenicity in humans (*44*).

Protection of newborn baboons from pertussis was achieved by antibody administration 1 day after birth and 5 weeks before pertussis infection. When anti-PTx antibodies were systemically present, leukocytosis and virtually all clinical signs of pertussis were mitigated, conclusively demonstrating the ability of neutralizing anti-PTx antibodies to prevent the signs and symptoms of pertussis. These data support passive immunization as a clinically viable strategy to complement maternal vaccination and protect high-risk newborns. Last, the data suggest that a serological correlate of protection from leukocytosis could be defined on the basis of neutralizing anti-PTx titers and that maternal immunization programs should strive to attain high serum neutralizing anti-PTx titers, likely via a PTg-only maternal booster vaccine, for the duration of the infant susceptible period.

## MATERIALS AND METHODS

### Experimental design

The objective of this controlled laboratory study was to assess the ability of antibody prophylaxis to diminish symptoms of pertussis in a neonatal model of disease. We compared two versions of the same antibody for pharmacokinetics and efficacy: the humanized anti-PTx antibody hu1B7 with a human IgG1 Fc and a variant with extended in vivo half-life due to three residue changes in the antibody Fc region (hu1B7-YTE). A mouse model was used to evaluate the role of Fc effector functions and potential impacts of hu1B7 prophylaxis on pertussis immunization. A recent *B. pertussis* clinical isolate, strain D420, was used to infect baboons and mice.

To determine the effect of hu1B7 on pertussis vaccination, Balb/c mice were administered a reverse chimera containing the human Fab portion of hu1B7 and a mouse IgG2a Fc region (r-ch1B7) or PBS and immunized 6 hours later with 1/50th human dose of acellular vaccine [JNIH-3, National Institute for Biological Standards and Control (NIBSC)]. Mice were boosted with same vaccine and dose 4 weeks later, with sera collected at several time points to monitor anti-Fha and anti-PTx titers.

A recently developed neonatal baboon model of pertussis was used to evaluate the pharmacokinetics and efficacy of neonatal prophylaxis with hu1B7 (*21*). Neonatal baboons were administered hu1B7 (eight animals), hu1B7-YTE (seven animals), or nothing (seven animals) the day after birth and then hand-raised. Animals were infected 4 to 5 weeks later and followed for signs of disease, including survival, leukocytosis, colonization, and clinical metrics. Baboons were randomly assigned to groups and animal caretakers, and laboratory technicians were not blinded. Four untreated animals became moribund and required euthanasia. The study was terminated ~80 days after birth, when WBC and bacterial colonization levels began to approach preinfection levels in control animals. After data were collected for the initial animals, power analysis was used to estimate that groups of seven animals would power the study at 97% to detect differences in the average peak WBC between treated and control groups using the *t* test with two-sided α = 0.05.

### Antibody variants and cloning

The unmodified human IgG1 antibody hu1B7 was used in all studies (*20*). Variants of hu1B7 were generated in the pJ602 and pJ607 plasmids (ATUM, Newark, CA) encoding hu1B7 heavy and light chains, respectively. A plasmid encoding hu1B7-YTE was generated by substituting three codons to generate the following amino acid changes: M272Y (ATG to TAC), S274T (AGC to ACC), and T276E (ACC to GAG) (*24*) and synthesized by ATUM. The aglycosylated antibody hu1B7-N297A was generated with mutagenic overlap polymerase chain reaction primers to create N297A (AAC to GCC). The r-ch1B7 antibody was created by replacing the human constant domains in the hu1B7 plasmids with those encoding the murine Igκ (GenBank AAA39012.1) and IgG2a (GenBank BAC44883.1) constant domains.

### Protein production and purification

Experiments with hu1B7 used the Catalent (Somerset, NJ) large-scale antibody preparation previously described (*20*). A large-scale hu1B7-YTE preparation was produced by ATUM using transient Chinese hamster ovary (CHO) cell transfection, followed by protein A chromatography and buffer exchange into PBS. The hu1B7 and hu1B7-YTE lots were similar in terms of antibody binding activity, percent monomeric protein, and endotoxin levels. Small-scale transient CHO transfection was used to produce r-ch1B7 and hu1B7-N297A as described (*20*). Purified PTx and Fha were obtained from List Labs and Enzo Life Sciences, respectively.

### Mouse and baboon serology

Indirect PTx and Fha ELISAs were performed on mouse and baboon serum as described (*20*). For antidrug antibody detection in baboon serum, a similar ELISA protocol was used with the exception of coating the plate with hu1B7 Fab (1 μg/ml) in PBS and use of goat anti-human Fc–horseradish peroxidase (Thermo Fisher Scientific) as the secondary antibody. For these ELISAs, a 1:50 dilution of each serum sample was analyzed in duplicate, with every serum sample from a single baboon on the same ELISA plate alongside a midrange control serum sample on each plate for normalization. Frozen aliquots of hu1B7, hu1B7-YTE, or r-ch1B7, previously found to equate to 9 ± 3 IU/μg hu1B7 using the World Health Organization (WHO) 06/142 standard (*20*), were used to standardize each PTx ELISA plate. Similarly, high titer pertussis intravenous immunoglobulin (P-IVIG) (*15*) of known unit concentration relative to WHO 06/142 or polyclonal mouse anti-Fha (JNIH-11, NIBSC) was used to standardize Fha ELISAs.

### Bacterial strain and growth

*B. pertussis* strain D420 was used in all experiments, with details of growth as described (*20*).

### Ethics statement

All animal procedures were performed in a facility accredited by the Association for Assessment and Accreditation of Laboratory Animal Care International in accordance with protocols approved by University of Texas, Austin (nos. 2018-00092 and 2017-00258) and the University of Oklahoma Health Sciences Center (no. 15-115-IC) Animal Care and Use Committees and the principles outlined in the *Guide for the Care and Use of Laboratory Animals*.

### Mouse infection and immunization

Four-week-old Balb/c mice (*n* = 6) were given 200 μl of antibody (10 or 1 μg of hu1B7 or 1 μg of hu1B7-N297A) or PBS intraperitoneally. Two hours later, mice were anesthetized (60 mg/kg ketamine and 8 mg/kg xylazine) and challenged intranasally with 10^7^ *B. pertussis* (25 μl/nare). Mice were housed in a biosafety level–2 (BSL2) suite and monitored daily. On day 7, mice were weighed and euthanized by cardiac puncture. Blood was immediately transferred to EDTA tubes, and lungs were harvested. Red blood cells were lysed, and remaining cells were stained with anti-CD45 (BioLegend) and analyzed on a Fortessa cytometer with counting beads (Invitrogen) to determine WBC as described (*19*). The lungs were weighed, homogenized in a final 1-ml volume of PBS, serially diluted, and plated on Bordet-Gengou blood agar plates. After 3 days of growth at 37°C, colonies were enumerated.

To assess the impact of antibody prophylaxis on subsequent vaccination, 20 μg of r-ch1B7 antibody or PBS in 200 μl was administered intraperitoneally 6 hours before vaccination. Following an initial blood draw and at 4 weeks, mice were administered PBS or a 1/50th human dose of human acellular vaccine (JNIH-3, NIBSC) subcutaneously in 200 μl of sterile PBS. Whole blood was collected from the tail vein weekly. After 6 weeks, mice were euthanized by CO_2_ inhalation, and cervical dislocation and sera were collected by cardiac puncture.

### Baboons

Neonatal baboon studies were performed at the Oklahoma Baboon Research Resource at the University of Oklahoma Health Sciences Center, as described previously (*21*). Infant baboons were born to mothers in the specific pathogen–free colony and transferred to the nonhuman primate nursery, where they were hand-fed human baby formula (Similac) ad libitum. Once inoculated with *B. pertussis*, animals were housed in animal BSL2^+^ until the end of the study. All baboons were euthanized for the evaluation of pathology at the end of the study or when meeting the following criteria: extreme lethargy, considerable leukocytosis, and severe tachypnea associated with adverse lung sounds as determined by auscultation.

### Baboon antibody infusion and pertussis inoculation

Neonates meeting the following criteria were recruited into the study: normal gestational age (180 days ± 10), normal birth weight (~1.0 kg), and anti-Fha titer <9.2 IU/ml on the day of infection (95% confidence interval for all animals before infection), indicating the absence of prior exposure to *Bordetella* species that can confound subsequent experimental infection (Table 1) (*29*). Infant baboons in the experimental group were sedated on day 2 of life, and a 25-gauge Teflon intravenous catheter was placed into the saphenous vein. Antibody (10 mg/ml) was slowly infused over 5 to 10 min to provide a 40 mg/kg dose. All animals were challenged with *B. pertussis* between 4 and 6 weeks of age. Inoculums were prepared to a concentration of 10^8^ *B. pertussis* bacteria/ml and administered as previously described (*21*).

### Baboon evaluation

Following infusion of antibody, infants were observed twice daily for 7 days to monitor clinical reactions. Baboons were raised in the nursery until challenge (*45*). Following challenge, infants were observed twice daily with clinical condition and behaviors noted. Case notes were reviewed by six blinded researchers, and an overall clinical score based on severity and duration of symptoms was assigned to each animal independently by each researcher. For biological sample collection, baboons were anesthetized with ketamine twice weekly. Whole blood was collected to monitor the circulating WBC count by complete blood count and PTx/ Fha titers by ELISA. Each nasal cavity was flushed with 0.5 ml of PBS using a 22-gauge/3.2-cm intravenous catheter, the two recovered nasopharyngeal washes combined before plating 50 μl in duplicate on Regan-Lowe plates, with *B. pertussis* CFU recorded after 4 to 5 days growth at 37°C.

### Statistical analyses

The means ± SD were determined for all appropriate data. For the murine and baboon challenge experiments, one-way analysis of variance (ANOVA) with Tukey’s simultaneous test with *P* values was used to determine statistical significance between groups. Antibody half-life and antibody concentration at infection for hu1B7 and hu1B7-YTE were compared using a two-tailed *t* test with unequal variances.

## Supplementary Material

http://advances.sciencemag.org/cgi/content/full/6/6/eaay9258/DC1

Download PDF

Neutralization of pertusis toxin by a single antibody prevents clinical pertusis in neonatal baboons

## SUPPLEMENTARY MATERIALS

Supplementary material for this article is available at http://advances.sciencemag.org/cgi/content/full/6/6/eaay9258/DC1 Fig. S1. Variants of antibody 1B7 bind PTx equivalently by ELISA. Fig. S2. Dosing hu1B7 in the mouse challenge model. Fig. S3. Temporal data for WBC count and bacterial colonization for each treatment group. Fig. S4. Temporal correlation of anti-PTx and WBC counts for each baboon. Fig. S5. Temporal correlation of anti-PTx and antidrug antibody titers for each treated baboon. Fig. S6. Pearson correlation of antidrug antibody titers and anti-PTx titers. Fig. S7. Presence of 1B7 does not interfere with anti-PTx responses due to vaccination. Table S1. Baboon raw data.

## Appendix

View/request a protocol for this paper from *Bio-protocol*.
