# Supplementary material for: Neutralization of pertussis toxin by a single antibody prevents clinical pertussis in neonatal baboons
Source: Sci Adv. 2020 Feb 5;6(6):eaay9258. doi: 10.1126/sciadv.aay9258 (PMC7002138; doi:10.1126/sciadv.aay9258)
Supplement: http://advances.sciencemag.org/cgi/content/full/6/6/eaay9258/DC1 [file supp_6_6_eaay9258__index.html]

Science Advances | Science AdvancesAAASSearchScience AdvancesMenu

## Supplementary Materials

**This PDF file includes:**

- Fig. S1. Variants of antibody 1B7 bind PTx equivalently by ELISA.
- Fig. S2. Dosing hu1B7 in the mouse challenge model.
- Fig. S3. Temporal data for WBC count and bacterial colonization for each treatment group.
- Fig. S4. Temporal correlation of anti-PTx and WBC counts for each baboon.
- Fig. S5. Temporal correlation of anti-PTx and antidrug antibody titers for each treated baboon.
- Fig. S6. Pearson correlation of antidrug antibody titers and anti-PTx titers.
- Fig. S7. Presence of 1B7 does not interfere with anti-PTx responses due to vaccination.
- Table S1. Baboon raw data.

Download PDF

**Files in this Data Supplement:**

- Adobe PDF - aay9258\_SM.pdf
