# Supplementary material for: Neutralization of pertussis toxin by a single antibody prevents clinical pertussis in neonatal baboons
Source: Sci Adv. 2020 Feb 5;6(6):eaay9258. doi: 10.1126/sciadv.aay9258 (PMC7002138; doi:10.1126/sciadv.aay9258)
Supplement: Download PDF [file aay9258_SM.pdf]

Science Advances
AAAS

advances.sciencemag.org/cgi/content/full/6/6/eaay9258/DC1

Supplementary Materials for

Neutralization of pertussis toxin by a single antibody prevents clinical pertussis in neonatal baboons

Annalee W. Nguyen, Andrea M. DiVenere, James F. Papin, Sheila Connelly, Michael Kaleko, Jennifer A. Maynard*

*Corresponding author. Email: maynard@che.utexas.edu

Published 5 February 2020, *Sci. Adv.* **6**, eaay9258 (2020)
DOI: 10.1126/sciadv.aay9258

This PDF file includes:

Fig. S1. Variants of antibody 1B7 bind PTx equivalently by ELISA.
Fig. S2. Dosing hu1B7 in the mouse challenge model.
Fig. S3. Temporal data for WBC count and bacterial colonization for each treatment group.
Fig. S4. Temporal correlation of anti-PTx and WBC counts for each baboon.
Fig. S5. Temporal correlation of anti-PTx and antidrug antibody titers for each treated baboon.
Fig. S6. Pearson correlation of antidrug antibody titers and anti-PTx titers.
Fig. S7. Presence of 1B7 does not interfere with anti-PTx responses due to vaccination.
Table S1. Baboon raw data.

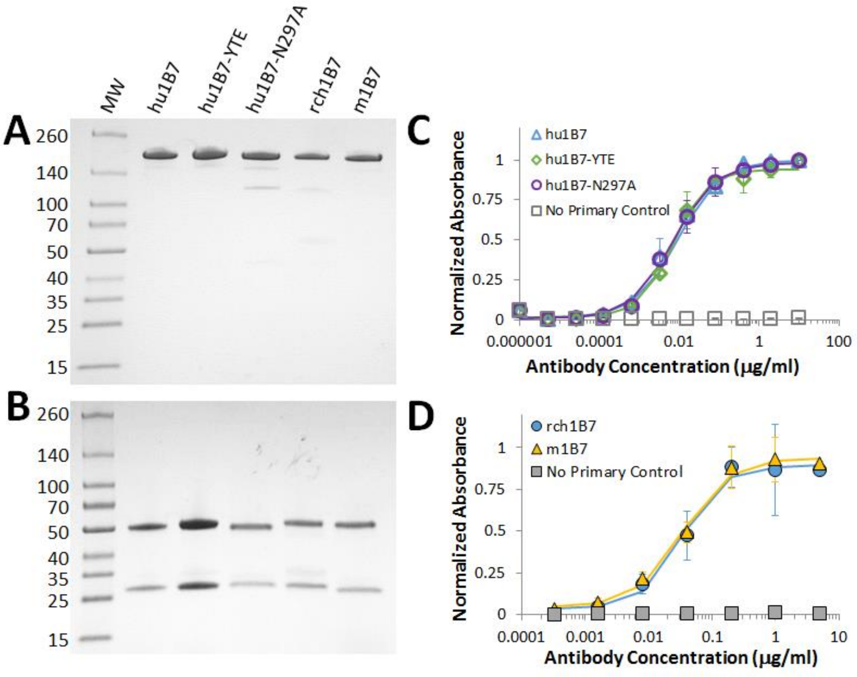

Fig. S1. Variants of antibody 1B7 bind PTx equivalently by ELISA. Variants of 1B7 were compared by (**A**) nonreducing and (**B**) reducing SDS-PAGE. Purified antibodies with (**C**) human or (**D**) mouse constant regions were compared for binding to a PTx coated plate by ELISA with detection by anti-human Fc or anti-mouse Fc antibodies, respectively. In (**C**), the average of duplicate hu1B7 data are in blue triangles, hu1B7-YTE are in green diamonds, hu1B7-N297A are in purple circles, and no primary control data are in grey squares. For (D) the average of quadruplicate r-ch1B7 data are in blue circles, m1B7 data are in yellow triangles, and no primary control data are in grey squares. Fits are shown as lines in the color of the data.

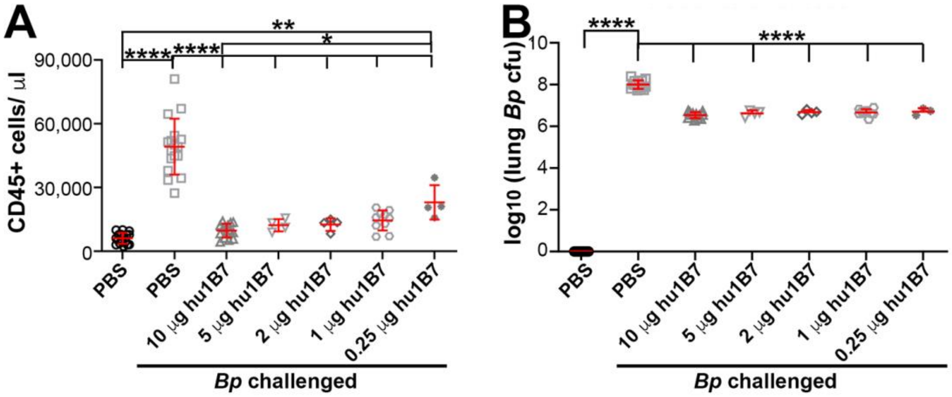

Fig. S2. Dosing hu1B7 in the mouse challenge model. Four-week-old Balb/c mice were administered antibody or PBS intraperitoneally in a 200 μl volume two hours prior to infection with 10^7 cfu *B. pertussis* strain D420. Infection severity was assessed after seven days by (**A**) CD45+ leukocytosis (WBC) and (**B**) *B. pertussis* lung colonization (no colonies were recovered for PBS-treated, uninfected mice). Data shown were pooled from three separate experiments, each including uninfected controls and infected controls treated with PBS or 10 μg hu1B7, with each group comprising 4-6 mice. Mean and standard deviation for each treatment are shown in red; significance was determined by two-way ANOVA with Tukey HSD with * $p<0.05$, ** $p<0.01$, **** $p<0.0001$.

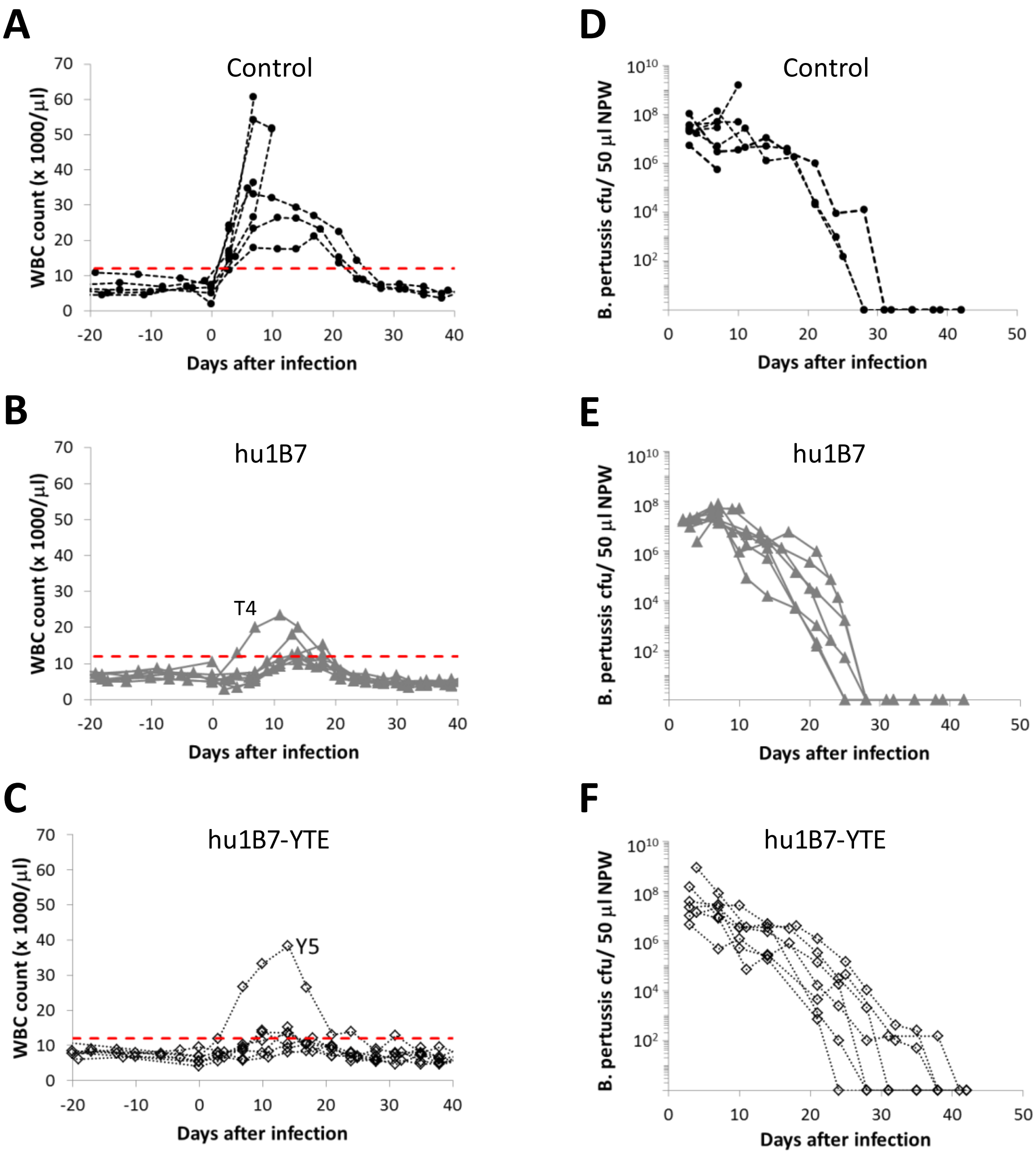

Fig. S3. Temporal data for WBC count and bacterial colonization for each treatment group. The WBC counts are shown for (**A**) control, (**B**) hu1B7-treated and (**C**) hu1B7-YTE treated animals. The dashed red line shows the 95% confidence interval for WBC variation observed before infection; WBC counts above this line are defined as elevated. The number of *B. pertussis* colonies recovered from nasopharyngeal wash fluid are shown for (**D**) controls, (**E**) hu1B7-treated and (**F**) hu1B7-YTE treated animals. Line and marker styles are consistent with **Figure 3C** in the main text.

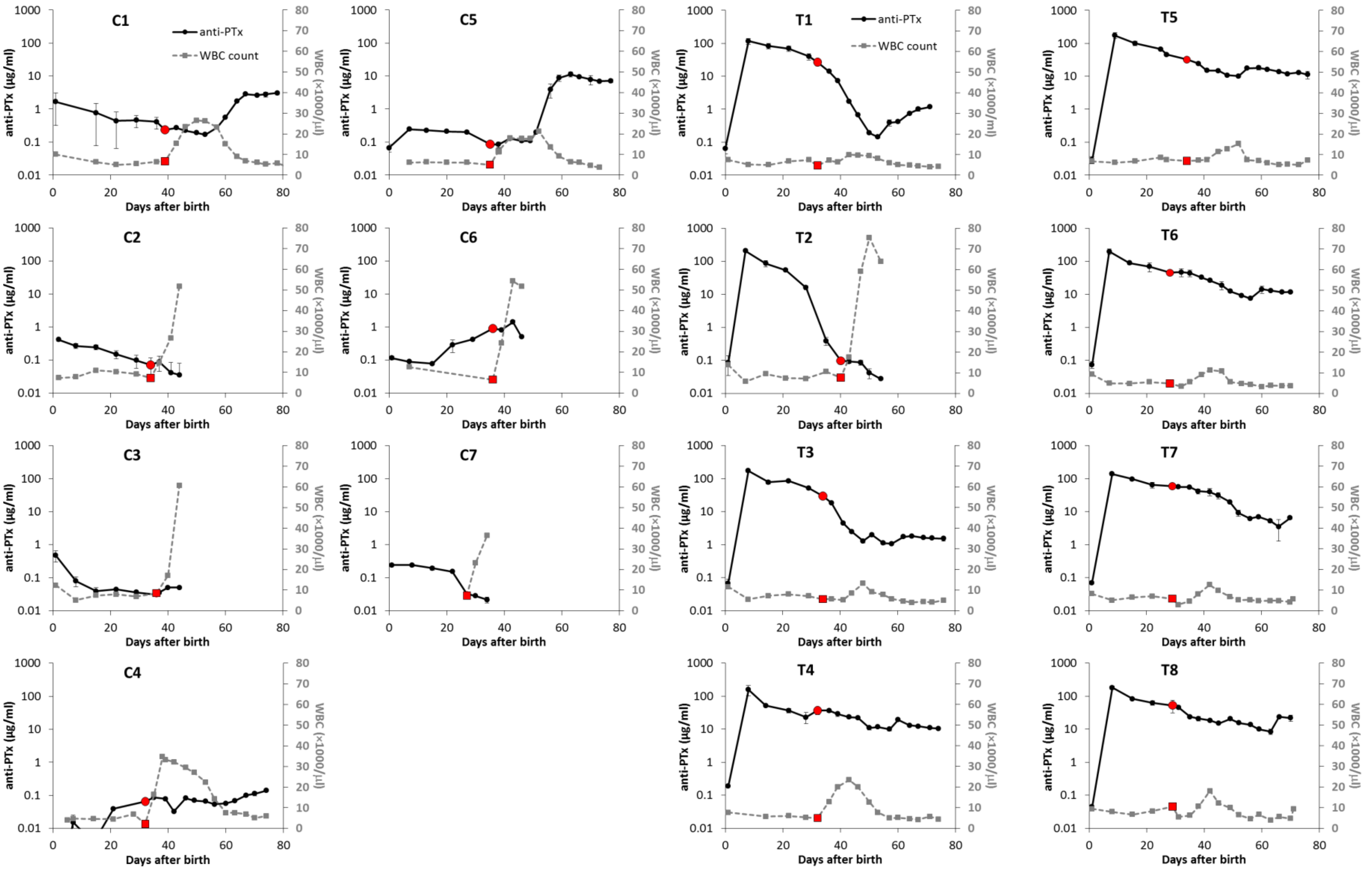
C1
C5
T1
T5
C2
C6
T2
T6
C3
C7
T3
T7
C4
T4
T8
anti-PTx
WBC count
anti-PTx (µg/ml)
WBC (×1000/µl)
Days after birth

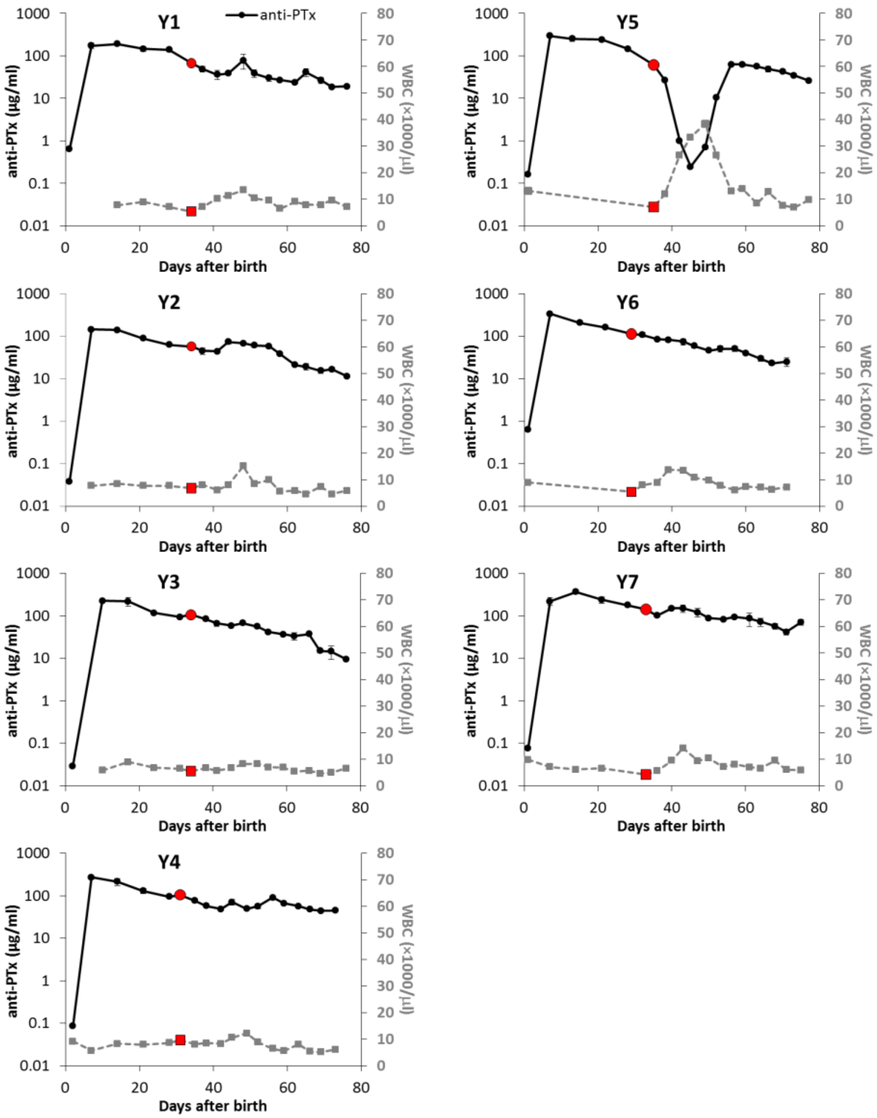

Fig. S4. Temporal correlation of anti-PTx and WBC counts for each baboon. Anti-PTx antibody titers were measured for each baboon (left vertical axis, black line and circles) and WBC count was determined for each point (right vertical axis, grey line and squares). In each data set, the day the baboon was infected with *B. pertussis* is identified by a red marker.

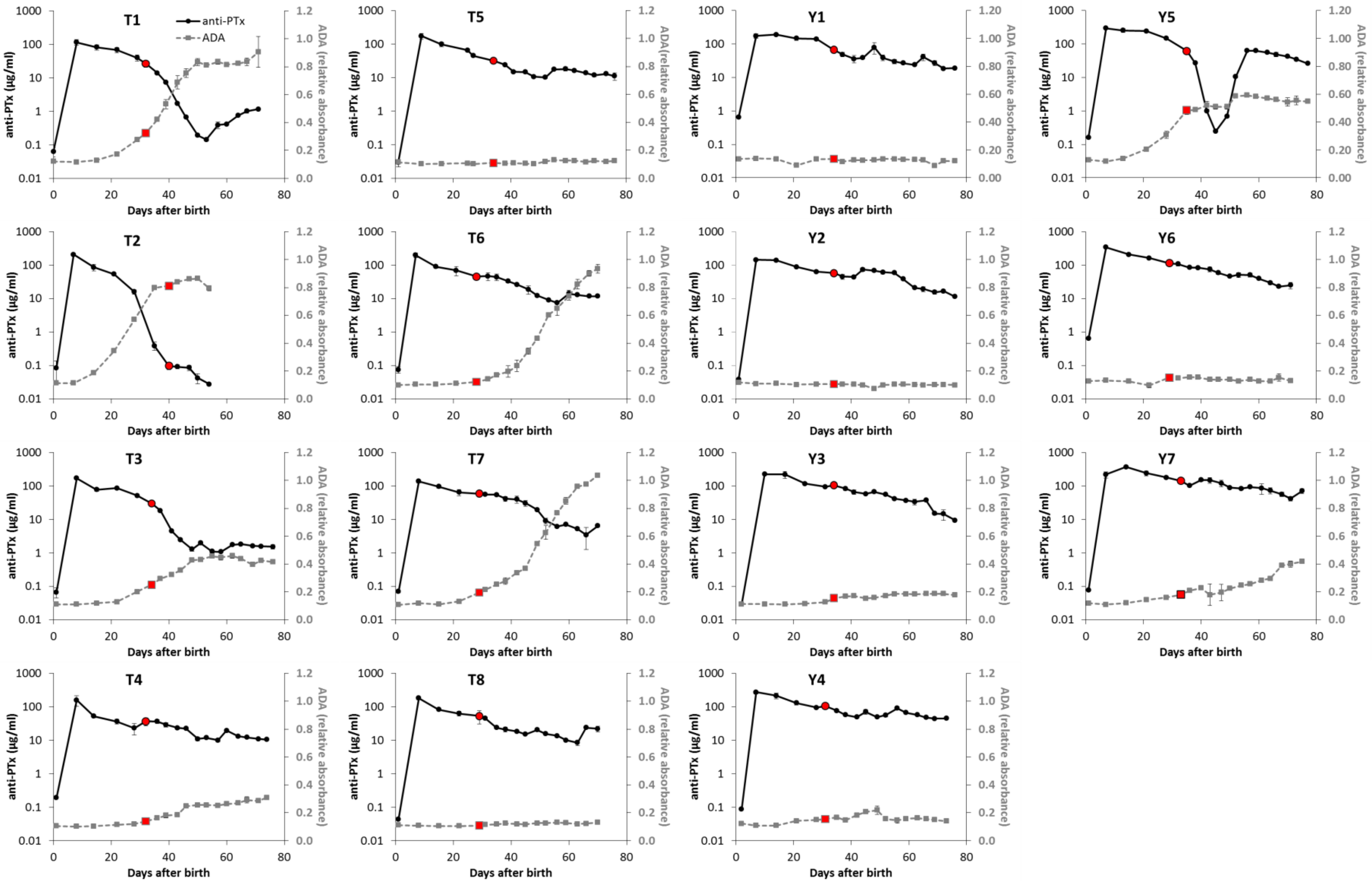

Fig. S5. Temporal correlation of anti-PTx and antidrug antibody titers for each treated baboon. Titers were measured for each treated baboon for antibodies binding PTx (left vertical axis, black line and circles) and antibodies binding the hu1B7 Fab (right vertical axis, grey line and squares). In each data set, the day the baboon was infected with *B. pertussis* is identified by a red marker.

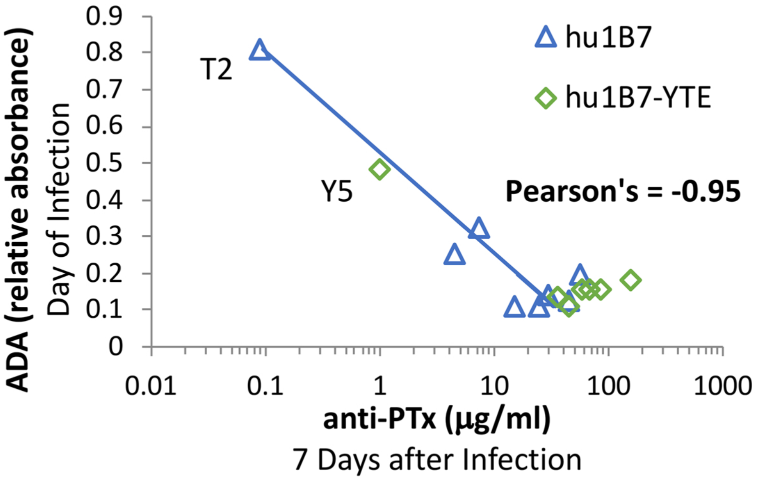

Fig. S6. Pearson correlation of antidrug antibody titers and anti-PTx titers. A strong correlation exists between anti-PTx antibody titer seven days after infection and day of infection anti-drug antibody titer (Pearson's correlation of -0.95 for hu1B7 treated animals). Data from animals treated with hu1B7 is shown in blue triangles and data from animals treated with hu1B7-YTE is shown in green diamonds.

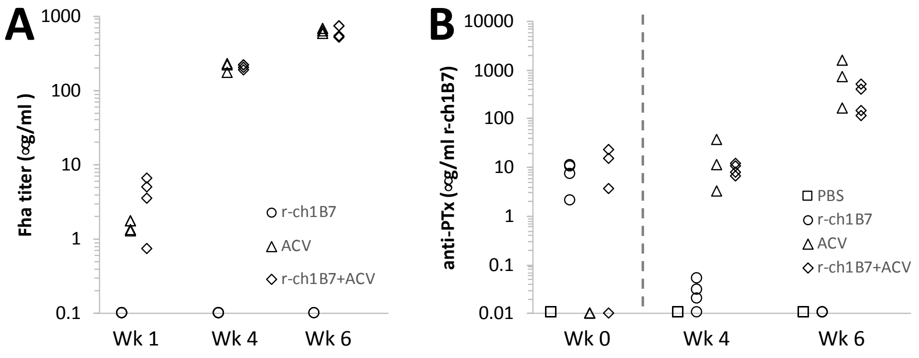

Fig. S7. Presence of 1B7 does not interfere with anti-PTx responses due to vaccination. Mice were administered 20 µg r-ch1B7 (human binding site + mouse Fc), immunized 24 hrs later with 1/50th human dose and boosted four weeks later. The (**A**) anti-Fha and (**B**) anti-PTx serum titers were monitored weekly for six weeks. Control mice included those only administered r-ch1B7 to monitor the titer of this specific antibody versus the polyclonal endogenous mouse response. No differences were observed in titers of immunized mice immunized based on the presence of r-ch1B7. Shown are data for individual mice; use of purified r-ch1B7 or a mouse anti-Fha antibody as a reference allowed for quantification of serum antibody titers and standardization between plates.

Supplementary Tables

Table S1. Baboon raw data. Data from each baboon's health records are compiled here. For each measurement, the average and standard deviation of values for all control baboons prior to infection was determined. Data falling outside of the 95% confidence interval for these values are highlighted in grey; *nd*, not determined.

Baboon C1

Age days	Challenge Day days	WBC Total x10^3/µl	WBC Neut x10^3/µl	WBC Lymph x10^3/µl	WBC Mono x10^3/µl	WBC Eosin x10^3/µl	WBC Baso x10^3/µl	RBC x10^6/µl	HGB g/dL	HCT %	PLT x10^3/µl	MCV fL	MCH pg	MCHC %	Retic %	Weight kg	Avg Temp °F	Avg HR /min	Avg Resp /min	serum anti-FHA IU/ml	+/-	serum anti-PTx µg/ml	+/-	serum ADA rel abs	+/-	NPW anti-PTx ng/ml	+/-
1	-38	10.08	7.38	1.62	1.04	0.02	0.02	2.90	11.8	27	231	94	40.7	43.4	2.86	0.90	nd	nd	nd	6.5	0.9	1.7	1.3	nd	nd	nd	nd
15	-24	6.33	3.13	2.43	0.71	0.05	0.01	3.76	11.3	33	375	88	30.1	34.1	3.67	1.10	nd	nd	nd	4.0	0.4	0.8	0.7	nd	nd	nd	nd
22	-17	5.02	1.49	2.83	0.67	0.03	0.00	4.4	11.5	38	341	86	26.1	30.4	3.29	nd	nd	nd	nd	2.6	0.3	0.4	0.4	nd	nd	nd	nd
29	-10	5.44	1.68	3.21	0.51	0.03	0.01	5.16	13.0	44	109	85	25.2	29.6	2.5	nd	98.2	nd	nd	3.2	1.1	0.5	0.2	nd	nd	nd	nd
36	-3	6.48	1.01	4.59	0.83	0.04	0.01	5.39	13.7	45	171	83	25.4	30.5	1.35	nd	nd	nd	nd	1.8	0.1	0.4	0.2	nd	nd	nd	nd
39	0	6.64	1.17	4.74	0.68	0.04	0.01	4.73	12.8	39	304	83	27.1	32.5	2.31	1.44	96.8	174	62	1.4	0.3	0.2	0.0	nd	nd	nd	nd
43	4	15.40	1.49	12.6	1.12	0.17	0.00	4.92	12.5	41	294	83	25.4	30.8	1.99	1.48	99.6	230	66	2.3	0.3	0.3	0.0	nd	nd	0.2	0.1
46	7	23.31	1.96	19.6	1.54	0.21	0.00	4.77	12.0	40	298	83	25.2	30.4	3.07	1.60	99.4	239	87	1.3	0.0	0.2	0.0	nd	nd	nd	nd
50	11	26.51	1.91	23.0	1.51	0.13	0.00	5.33	13.3	44	339	83	25.0	30.0	2.66	1.59	98.9	218	87	2.3	0.2	0.2	0.0	nd	nd	nd	nd
53	14	26.2	2.28	21.9	1.89	0.13	0.00	5.28	13.1	43	331	81	24.8	30.8	1.99	1.64	99	228	87	2.3	0.0	0.2	0.0	nd	nd	nd	nd
57	18	23.22	5.99	16.0	1.18	0.09	0.00	5.13	12.8	42	339	83	25.0	30.2	2.31	1.66	96.8	174	63	4.3	0.2	0.3	0.0	nd	nd	nd	nd
60	21	15.16	1.93	11.8	1.36	0.06	0.00	5.15	12.7	42	273	82	24.7	30.2	1.56	1.74	99.1	184	63	2.8	0.0	0.6	0.0	nd	nd	nd	nd
64	25	8.95	1.54	6.33	1.05	0.04	0.00	4.94	12.2	40	267	82	24.7	30.3	2.88	1.79	99.2	211	52	7.8	0.0	1.7	0.0	nd	nd	nd	nd
67	28	6.87	2.39	3.58	0.88	0.02	0.00	5.17	12.7	42	257	81	24.6	30.2	2.56	1.80	98.4	181	84	16.1	2.7	2.8	0.2	nd	nd	nd	nd
71	32	6.16	1.22	4.26	0.63	0.04	0.01	5.23	12.9	42	247	81	24.7	30.6	2.01	1.85	99.3	200	66	40.4	0.0	2.6	0.1	nd	nd	nd	nd
74	35	5.24	1.03	3.67	0.51	0.03	0.00	5.06	12.5	40	289	80	24.7	30.9	1.87	1.90	99.4	210	69	30.1	8.8	2.8	0.5	nd	nd	nd	nd
78	39	5.82	1.28	4.05	0.45	0.04	0.00	5.53	13.7	44	338	79	24.8	31.3	1.63	1.88	98.9	197	59	136.3	7.2	3.1	0.3	nd	nd	nd	nd
81	42	4.25	1.52	2.37	0.34	0.01	0.01	5.66	13.9	45	329	79	24.6	31.2	1.00	1.88	98.8	225	84	nd	nd	nd	nd	nd	nd	nd	nd

Baboon C2

Age (days)	Challenge Day (days)	WBC Total ($x10^3$/µl)	WBC Neut ($x10^3$/µl)	WBC Lymph ($x10^3$/µl)	WBC Mono ($x10^3$/µl)	WBC Eosin ($x10^3$/µl)	WBC Baso ($x10^3$/µl)	RBC ($x10^6$/µl)	HGB (g/dL)	HCT (%)	PLT ($x10^3$/µl)	MCV (fL)	MCH (pg)	MCHC (%)	Retic (%)	Weight (kg)	Avg Temp (°F)	Avg HR (/min)	Avg Resp (/min)	serum anti-FHA (IU/ml)	serum anti-FHA (+/-)	serum anti-PTx (µg/ml)	serum anti-PTx (+/-)	serum ADA (rel abs)	serum ADA (+/-)	NPW anti-PTx (ng/ml)	NPW anti-PTx (+/-)
2	-32	7.38	3.62	2.94	0.77	0.0	0.0	5.14	16.0	51.8	460	101.0	31.1	30.9	1.6	nd	nd	nd	nd	1.3	0.0	0.4	0.0	nd	nd	nd	nd
8	-26	7.80	3.06	3.91	0.81	0.0	0.0	4.94	14.9	46.6	594	94.3	30.2	32.0	0.3	nd	nd	nd	nd	1.1	0.0	0.3	0.0	nd	nd	nd	nd
15	-19	10.97	4.08	5.98	0.87	0.0	0.0	4.70	13.7	43.6	676	92.8	29.1	31.4	0.9	nd	nd	nd	nd	0.9	0.1	0.2	0.0	nd	nd	nd	nd
22	-12	10.40	4.34	5.42	0.61	0.0	0.0	4.42	12.4	40.0	450	90.5	28.1	31.0	2.6	nd	nd	nd	nd	1.2	0.1	0.2	0.0	nd	nd	nd	nd
29	-5	9.30	3.14	5.62	0.50	0.0	0.0	4.60	12.6	40.3	495	87.6	27.4	31.3	2.6	nd	nd	nd	nd	0.9	0.1	0.1	0.0	nd	nd	nd	nd
34	0	7.31	4.03	3.05	0.23	0.0	0.0	4.32	11.7	37.0	455	85.6	27.1	31.6	2.1	nd	99.1	nd	nd	0.8	0.1	0.1	0.0	nd	nd	nd	nd
37	3	14.73	3.48	10.8	0.34	0.1	0.0	4.21	11.1	35.0	496	83.1	26.4	31.7	1.8	1.16	97.8	178	50	0.3	0.0	0.1	0.0	nd	nd	0.03	0.01
41	7	26.68	6.72	18.9	0.91	0.2	0.0	4.25	11.1	35.2	429	82.8	26.1	31.5	2.0	1.22	98.5	221	99	0.4	0.0	0.0	0.0	nd	nd	nd	nd
44	10	51.81	21.6	26.3	3.57	0.4	0.0	4.21	10.7	35.3	454	83.8	25.4	30.3	1.6	nd	99.0	199	74	0.6	0.0	0.0	0.0	nd	nd	nd	nd

Baboon C3

Age (days)	Challenge Day (days)	WBC Total ($x10^3$/µl)	WBC Neut ($x10^3$/µl)	WBC Lymph ($x10^3$/µl)	WBC Mono ($x10^3$/µl)	WBC Eosin ($x10^3$/µl)	WBC Baso ($x10^3$/µl)	RBC ($x10^6$/µl)	HGB (g/dL)	HCT (%)	PLT ($x10^3$/µl)	MCV (fL)	MCH (pg)	MCHC (%)	Retic (%)	Weight (kg)	Avg Temp (°F)	Avg HR (/min)	Avg Resp (/min)	serum anti-FHA (IU/ml)	serum anti-FHA (+/-)	serum anti-PTx (µg/ml)	serum anti-PTx (+/-)	serum ADA (rel abs)	serum ADA (+/-)	NPW anti-PTx (ng/ml)	NPW anti-PTx (+/-)
1	-36	12.33	8.38	2.93	0.83	0.2	0.0	4.31	13.4	38.5	253	89.3	31.1	34.8	3.2	nd	nd	nd	nd	6.5	1.4	0.5	0.2	nd	nd	nd	nd
8	-29	5.10	1.35	3.31	0.42	0.0	0.0	4.51	13.5	40.8	417	90.5	29.9	33.1	1.3	nd	nd	nd	nd	5.9	0.7	0.1	0.0	nd	nd	nd	nd
15	-22	7.47	2.03	4.94	0.45	0.1	0.0	4.70	13.6	41.4	217	88.1	28.9	32.9	3.8	nd	nd	nd	nd	4.8	0.0	0.0	0.0	nd	nd	nd	nd
22	-15	8.01	2.15	5.31	0.47	0.1	0.0	4.65	13.0	40.5	282	87.1	28.0	32.1	3.6	nd	nd	nd	nd	2.2	0.2	0.0	0.0	nd	nd	nd	nd
29	-8	6.92	1.78	4.74	0.36	0.0	0.0	4.68	12.8	39.2	367	83.8	27.4	32.7	3.5	nd	nd	nd	nd	1.7	0.0	0.0	0.0	nd	nd	nd	nd
36	-1	8.53	1.95	6.05	0.51	0.0	0.0	5.38	14.4	45.2	202	84.0	26.8	31.9	1.7	nd	nd	nd	nd	1.6	0.2	0.0	0.0	nd	nd	nd	nd
37	0	nd	nd	nd	nd	nd	nd	nd	nd	nd	nd	nd	nd	nd	nd	1.22	98.9	189	51	1.4	0.1	0.0	0.0	nd	nd	nd	nd
40	3	17.13	2.84	13.6	0.62	0.1	0.0	5.06	13.4	41.6	279	82.2	26.5	32.2	0.7	1.24	98.8	190	93	1.9	0.2	0.1	0.0	nd	nd	0.1	0.1
44	7	56.89	21.0	30.0	5.66	0.3	0.0	4.64	12.2	38.6	340	83.2	26.2	31.5	1.7	1.2	97.3	215	82	0.8	0.0	0.1	0.0	nd	nd	nd	nd

Baboon C4

Age	Challenge Day	WBC Total	WBC Neut	WBC Lymph	WBC Mono	WBC Eosin	WBC Baso	RBC	HGB	HCT	PLT	MCV	MCH	MCHC	Retic	Weight	Avg Temp	Avg HR	Avg Resp	serum anti-FHA	+/-	serum anti-PTx	+/-	serum ADA	+/-	NPW anti-PTx	+/-
days	days	x10^3/µl	x10^3/µl	x10^3/µl	x10^3/µl	x10^3/µl	x10^3/µl	x10^6/µl	g/dL	%	x10^3/µl	fL	pg	%	%	kg	°F	/min	/min	IU/ml	+/-	µg/ml	+/-	rel abs	+/-	ng/ml	+/-
5	-27	4.03	2.29	1.33	0.33	0.0	0.0	5.41	15.8	49.3	291	91.1	29.2	32.0	0.6	nd	nd	nd	nd	0.4	0.0	0.0	0.0	nd	nd	nd	nd
7	-25	4.65	1.72	2.29	0.53	0.1	0.0	5.31	15.3	48.2	270	90.8	28.8	31.7	0.5	nd	nd	nd	nd	0.3	0.1	0.0	0.0	nd	nd	nd	nd
14	-18	4.61	1.96	2.23	0.33	0.1	0.0	4.87	13.8	41.0	404	84.2	28.3	33.7	1.8	nd	nd	nd	nd	0.4	0.0	0.0	0.0	nd	nd	nd	nd
21	-11	4.48	1.74	2.34	0.22	0.1	0.0	4.57	12.3	38.3	400	83.8	26.9	32.1	2.1	nd	nd	nd	nd	3.0	0.1	0.0	0.0	nd	nd	nd	nd
28	-4	6.9	2.61	3.89	0.29	0.1	0.0	4.96	12.6	40.8	462	82.3	25.4	30.9	3.0	nd	nd	nd	nd	nd	nd	nd	nd	nd	nd	nd	nd
32	0	2.09	0.83	1.12	0.13	0.0	0.0	4.57	11.2	35.7	415	78.1	24.5	31.4	2.1	1.15	99.1	157	54	4.0	1.1	0.1	0.0	nd	nd	nd	nd
35	3	16.34	4.93	10.8	0.46	0.1	0.0	4.45	10.9	34.3	408	77.1	24.5	31.8	1.2	1.20	99.8	171	71	4.5	0.1	0.1	0.0	nd	nd	0.0	0.0
39	7	33.24	19.1	12.2	1.63	0.2	0.1	4.24	9.7	32.2	377	75.9	22.9	30.1	1.1	1.12	100.5	178	114	3.6	0.1	0.1	0.0	nd	nd	nd	nd
42	10	32.15	16.6	13.1	2.38	0.1	0.1	4.41	9.8	32.7	563	74.1	22.2	30.0	1.3	1.11	99.8	214	109	4.8	2.5	0.0	0.0	nd	nd	nd	nd
46	14	29.39	14.9	12.6	1.65	0.1	0.0	4.71	10.1	33.9	333	72.0	21.4	29.8	1.3	1.09	97.9	nd	nd	17.4	1.1	0.1	0.0	nd	nd	nd	nd
49	17	27.04	12.2	13.3	1.22	0.2	0.0	4.47	9.4	31.1	233	69.6	21.0	30.2	2.4	1.15	98.7	nd	nd	43.3	0.7	0.1	0.0	nd	nd	nd	nd
53	21	22.39	11.6	8.84	1.72	0.2	0.0	4.66	9.9	32.6	194	70.0	21.2	30.4	3.5	1.20	98.6	nd	nd	212.7	0.9	0.1	0.0	nd	nd	nd	nd
56	24	14.26	7.33	5.60	1.10	0.2	0.0	4.78	10.1	32.5	338	68.0	21.1	31.1	4.2	1.19	98.5	nd	nd	651.0	14.8	0.1	0.0	nd	nd	nd	nd
60	28	7.52	3.29	3.18	0.76	0.3	0.0	4.78	9.8	32.6	353	68.2	20.5	30.1	2.5	1.22	97.9	nd	nd	1522.3	449.6	0.1	0.0	nd	nd	nd	nd
63	31	7.54	3.92	2.75	0.66	0.2	0.0	4.86	10.1	33.5	422	68.9	20.8	30.1	2.3	1.24	98.7	nd	nd	5526.2	224.4	0.1	0.0	nd	nd	nd	nd
67	35	6.82	4.33	1.92	0.49	0.1	0.0	5.09	10.6	34.7	375	68.2	20.8	30.5	2.2	1.27	98.9	nd	nd	4917.2	368.3	0.1	0.0	nd	nd	nd	nd
70	38	4.98	3.19	1.46	0.27	0.1	0.0	4.96	10.5	33.8	305	68.1	21.2	31.1	2.0	1.30	98.4	nd	nd	3548.1	83.8	0.1	0.0	nd	nd	nd	nd
74	42	5.97	3.52	2.06	0.28	0.1	0.0	5.21	11.2	36.7	288	70.4	21.5	30.5	2.6	1.33	99.4	nd	nd	3221.7	73.9	0.1	0.0	nd	nd	nd	nd

Baboon C5

Age<br>days	Challenge Day<br>days	WBC Total<br>x10³/µl	WBC Neut<br>x10³/µl	WBC Lymph<br>x10³/µl	WBC Mono<br>x10³/µl	WBC Eosin<br>x10³/µl	WBC Baso<br>x10³/µl	RBC<br>x10⁶/µl	HGB<br>g/dL	HCT<br>%	PLT<br>x10³/µl	MCV<br>fL	MCH<br>pg	MCHC<br>%	Retic<br>%	Weight<br>kg	Avg Temp<br>°F	Avg HR<br>/min	Avg Resp<br>/min	serum anti-FHA<br>IU/ml	+/-	serum anti-PTx<br>µg/ml	+/-	serum ADA<br>rel abs	+/-	NPW anti-PTx<br>ng/ml	+/-
0	-35	nd	nd	nd	nd	nd	nd	nd	nd	nd	nd	nd	nd	nd	nd	nd	nd	nd	nd	0.4	0.0	0.1	0.0	nd	nd	nd	nd
7	-28	6.08	2.83	2.51	0.65	0.1	0.0	5.75	16.7	50.6	344	88.0	29.0	33.0	0.4	nd	nd	nd	nd	0.4	0.0	0.2	0.0	nd	nd	nd	nd
13	-22	6.36	2.79	3.00	0.52	0.0	0.0	5.07	14.4	43.6	644	86.0	28.4	33.0	2.6	nd	nd	nd	nd	0.8	0.3	0.2	0.0	nd	nd	nd	nd
20	-15	6.07	2.01	3.47	0.45	0.1	0.0	4.91	13.7	41.1	367	83.7	27.9	33.3	4.2	nd	nd	nd	nd	1.0	0.1	0.2	0.0	nd	nd	nd	nd
27	-8	6.11	1.73	3.88	0.43	0.1	0.0	4.69	12.7	39.6	430	84.4	27.1	32.1	5.0	nd	nd	nd	nd	0.6	0.1	0.2	0.0	nd	nd	nd	nd
35	0	5.04	2.99	1.90	0.14	0.0	0.0	4.72	12.6	38.2	425	80.9	26.7	33.0	2.6	1.3	98.9	nd	nd	0.9	0.0	0.1	0.0	nd	nd	nd	nd
38	3	11.65	2.85	8.29	0.40	0.1	0.0	4.79	12.6	39.3	381	82.0	26.3	32.1	2.2	1.29	99.6	nd	nd	0.8	0.0	0.1	0.0	nd	nd	0.0	0.0
42	7	17.94	3.77	13.6	0.47	0.1	0.0	4.57	11.8	36.6	468	80.1	25.8	32.2	4.2	1.39	98.2	nd	nd	0.9	0.0	0.1	0.0	nd	nd	nd	nd
46	11	17.63	2.15	14.6	0.71	0.1	0.0	4.71	12.2	38.5	500	81.7	25.9	31.7	5.0	1.42	99.0	nd	nd	1.1	0.2	0.1	0.0	nd	nd	nd	nd
49	14	17.62	1.74	15.1	0.65	0.1	0.0	5.18	13.2	42.2	506	81.5	25.5	31.3	4.0	1.47	99.6	nd	nd	1.9	0.2	0.1	0.0	nd	nd	nd	nd
51	16	21.16	2.45	17.2	1.25	0.1	0.0	4.92	12.6	39.7	524	80.7	25.6	31.7	3.4	1.54	98.9	nd	nd	3.0	0.6	0.2	0.0	nd	nd	nd	nd
56	21	13.55	2.04	10.9	0.51	0.0	0.0	5.20	13.2	41.9	614	80.6	25.4	31.5	3.3	1.63	99.5	nd	nd	18.9	1.3	4.0	1.8	nd	nd	nd	nd
59	24	9.13	1.92	6.90	0.28	0.0	0.0	4.96	12.7	40.2	553	81.0	25.6	31.6	2.7	1.60	99.4	nd	nd	79.3	3.7	8.7	1.9	nd	nd	nd	nd
63	28	6.43	2.01	4.06	0.35	0.0	0.0	4.91	12.5	39.4	547	80.2	25.5	31.7	3.6	1.63	99.6	nd	nd	195.8	47.5	11.3	1.9	nd	nd	nd	nd
66	31	6.15	2.58	3.22	0.32	0.0	0.0	5.07	12.7	40.5	540	79.9	25	31.4	2.5	1.67	99.4	nd	nd	199.2	21.4	9.4	0.4	nd	nd	nd	nd
70	35	4.62	0.79	3.43	0.36	0.0	0.0	5.18	13.1	40.6	367	78.4	25.3	32.3	2.0	1.72	100.2	nd	nd	237.0	67.1	7.9	2.5	nd	nd	nd	nd
73	38	3.72	0.60	2.73	0.35	0.0	0.0	5.08	12.6	40.3	379	79.3	24.8	31.3	1.8	1.76	100.2	nd	nd	279.2	45.3	6.9	0.3	nd	nd	nd	nd
77	42	5.96	2.12	3.49	0.31	0.0	0.0	5.37	13.5	42.1	438	78.4	25.1	32.1	1.7	nd	98.25	176.0	32.0	326.3	90.6	7.2	0.9	nd	nd	nd	nd

Baboon C6

Age days	Challenge Day days	WBC Total $x10^3$/µl	WBC Neut $x10^3$/µl	WBC Lymph $x10^3$/µl	WBC Mono $x10^3$/µl	WBC Eosin $x10^3$/µl	WBC Baso $x10^3$/µl	RBC $x10^6$/µl	HGB g/dL	HCT %	PLT $x10^3$/µl	MCV fL	MCH pg	MCHC %	Retic %	Weight kg	Avg Temp °F	Avg HR /min	Avg Resp /min	serum anti-FHA IU/ml	serum anti-FHA +/-	serum anti-PTx µg/ml	serum anti-PTx +/-	serum ADA rel abs	serum ADA +/-	NPW anti-PTx ng/ml	NPW anti-PTx +/-
1	-35	nd	nd	nd	nd	nd	nd	nd	nd	nd	nd	nd	nd	nd	nd	nd	nd	nd	nd	0.6	0.0	0.1	0.0	nd	nd	nd	nd
7	-29	12.65	8.12	3.59	0.78	0.1	0.0	5.55	17	51	383	92.6	30.1	32.5	0.3	nd	nd	nd	nd	1.0	0.1	0.1	0.0	nd	nd	nd	nd
15	-21	nd	nd	nd	nd	nd	nd	nd	nd	nd	nd	nd	nd	nd	nd	nd	nd	nd	nd	1.0	0.3	0.1	0.0	nd	nd	nd	nd
22	-14	nd	nd	nd	nd	nd	nd	nd	nd	nd	nd	nd	nd	nd	nd	nd	nd	nd	nd	1.9	0.3	0.3	0.1	nd	nd	nd	nd
29	-7	nd	nd	nd	nd	nd	nd	nd	nd	nd	nd	nd	nd	nd	nd	nd	nd	nd	nd	2.9	0.3	0.4	0.0	nd	nd	nd	nd
36	0	6.58	3.01	3.32	0.13	0.1	0.0	4.70	13	38	504	81.5	27.2	33.4	1.0	1.36	101.1	nd	nd	3.8	0.6	0.9	0.1	nd	nd	nd	nd
39	3	24.25	7.54	15.5	0.78	0.4	0.0	4.55	12	38	420	83.1	27.0	32.5	1.2	1.44	100.1	nd	nd	2.0	0.1	0.8	0.1	nd	nd	0.0	0.0
43	7	54.27	15.0	35.1	3.53	0.7	0.0	4.94	13	41	540	83.8	25.9	30.9	2.0	1.405	100.2	nd	nd	2.3	0.4	1.4	0.2	nd	nd	nd	nd
46	10	51.67	18.2	24.7	8.11	0.6	0.1	5.24	13	42	94	80.5	25.2	31.3	1.0	1.385	98.0	nd	nd	1.0	0.0	0.5	0.0	nd	nd	nd	nd

Baboon C7

Age days	Challenge Day days	WBC Total $x10^3$/µl	WBC Neut $x10^3$/µl	WBC Lymph $x10^3$/µl	WBC Mono $x10^3$/µl	WBC Eosin $x10^3$/µl	WBC Baso $x10^3$/µl	RBC $x10^6$/µl	HGB g/dL	HCT %	PLT $x10^3$/µl	MCV fL	MCH pg	MCHC %	Retic %	Weight kg	Avg Temp °F	Avg HR /min	Avg Resp /min	serum anti-FHA IU/ml	serum anti-FHA +/-	serum anti-PTx µg/ml	serum anti-PTx +/-	serum ADA rel abs	serum ADA +/-	NPW anti-PTx ng/ml	NPW anti-PTx +/-
1	-26	nd	nd	nd	nd	nd	nd	nd	nd	nd	nd	nd	nd	nd	nd	nd	nd	nd	nd	1.1	0.0	0.2	0.0	nd	nd	nd	nd
8	-19	9.06	2.75	5.82	0.34	0.1	0.1	5.2	15.9	47.8	636	92.5	30.8	33.3	0.3	nd	nd	nd	nd	0.8	0.1	0.2	0.0	nd	nd	nd	nd
15	-12	7.96	2.90	4.72	0.26	0.1	0.0	4.4	13.2	39.7	673	89.4	29.7	33.2	1.9	nd	nd	nd	nd	0.5	0.0	0.2	0.0	nd	nd	nd	nd
22	-5	nd	nd	nd	nd	nd	nd	nd	nd	nd	nd	nd	nd	nd	nd	nd	nd	nd	nd	0.4	0.0	0.2	0.0	nd	nd	nd	nd
27	0	7.41	5.19	2.08	0.12	0.0	0.0	3.9	11.1	32.2	525	81.7	28.2	34.5	1.2	1.11	100.4	135.0	33.0	0.3	0.0	0.0	0.0	nd	nd	nd	nd
30	3	23.27	4.93	17.6	0.56	0.1	0.0	4.1	11.4	34.2	382	84.2	28.1	33.3	1.63	1.17	98.4	173.5	54.0	0.4	0.0	0.0	0.0	nd	nd	0.0	0.1
34	7	36.42	5.86	29.5	0.76	0.3	0.0	4.3	11.8	36.4	222	84.5	27.4	32.4	3.25	1.16	nd	nd	nd	0.3	0.0	0.0	0.0	nd	nd	nd	nd

Baboon T1

Age	Challenge Day	WBC Total	WBC Neut	WBC Lymph	WBC Mono	WBC Eosin	WBC Baso	RBC	HGB	HCT	PLT	MCV	MCH	MCHC	Retic	Weight	Avg Temp	Avg HR	Avg Resp	serum anti-FHA		serum anti-PTx		serum ADA		NPW anti-PTx	
days	days	x10³/µl	x10³/µl	x10³/µl	x10³/µl	x10³/µl	x10³/µl	x10⁶/µl	g/dL	%	x10³/µl	fL	pg	%	%	kg	°F	/min	/min	IU/ml	+/-	µg/ml	+/-	rel abs	+/-	ng/ml	+/-
0	-32	7.39	6.21	0.69	0.48	0.0	0.0	4.22	14.5	41.8	313	99.1	34.4	34.7	2.8	1.0	99.8	174	60	1.5	0.8	0.1	0.0	0.12	0.00	nd	nd
8	-24	5.18	2.00	2.59	0.57	0.0	0.0	4.22	15.0	41.5	322	98.3	35.5	36.1	0.6	1.1	nd	nd	nd	1.2	0.6	116.0	21.6	0.12	0.00	nd	nd
15	-17	5.05	1.30	3.21	0.51	0.0	0.0	4.77	14.8	46.2	394	96.9	31.0	32.0	2.2	nd	nd	nd	nd	1.3	0.5	83.0	15.2	0.13	0.00	nd	nd
22	-10	6.72	2.09	3.94	0.63	0.1	0.0	4.63	13.7	43.5	380	94.0	29.6	31.5	2.6	nd	nd	nd	nd	1.1	0.2	69.1	12.4	0.17	0.00	nd	nd
29	-3	7.42	1.67	5.29	0.41	0.1	0.0	4.52	13.0	41.0	416	90.7	28.8	31.7	4.0	nd	nd	nd	nd	1.3	0.0	40.0	8.8	0.28	0.00	nd	nd
32	0	4.78	1.46	3.02	0.26	0.0	0.0	4.02	12.4	36.8	362	91.5	30.8	33.7	4.3	1.55	97.2	158	52	0.9	0.1	26.6	6.1	0.33	0.00	nd	nd
36	4	7.21	1.98	4.79	0.36	0.1	0.0	4.34	12.1	38.7	327	89.2	27.9	31.3	2.5	1.60	99.4	204	54	0.6	0.1	14.1	0.4	0.42	0.01	5.9	0.5
39	7	6.33	1.04	4.96	0.25	0.1	0.0	4.41	12.4	38.6	427	87.5	28.1	32.1	3.9	1.63	99.0	172	56	1.4	0.6	7.3	0.5	0.53	0.03	nd	nd
43	11	9.92	1.56	8.01	0.28	0.1	0.0	4.68	12.9	41.0	514	87.6	27.6	31.5	3.3	1.68	98.5	191	52	0.5	0.1	1.7	0.1	0.69	0.05	nd	nd
46	14	9.71	1.44	7.96	0.25	0.0	0.0	4.78	12.9	41.1	448	86.0	27.0	31.4	1.7	1.74	98.7	177	57	1.4	0.2	0.7	0.0	0.75	0.03	nd	nd
50	18	9.38	1.63	7.35	0.35	0.0	0.0	4.66	12.5	39.8	375	85.4	26.8	31.4	1.6	1.71	98.6	162	73	3.3	0.3	0.2	0.0	0.83	0.03	nd	nd
53	21	8.09	0.81	6.91	0.33	0.0	0.0	4.41	11.8	37.8	374	85.7	26.8	31.2	1.4	1.83	98.8	192	56	4.5	0.2	0.1	0.0	0.81	0.00	nd	nd
57	25	6.00	0.90	4.83	0.26	0.0	0.0	4.70	12.6	39.8	422	84.7	26.8	31.7	2.4	1.82	98.3	148	58	7.0	0.2	0.4	0.1	0.84	0.02	nd	nd
60	28	5.05	1.44	3.35	0.23	0.0	0.0	4.87	12.9	40.8	470	83.8	26.5	31.6	1.5	1.85	97.9	173	52	28.6	0.1	0.4	0.0	0.82	0.01	nd	nd
64	32	4.80	0.76	3.73	0.29	0.0	0.0	5.15	13.7	43.0	411	83.5	26.6	31.9	1.5	1.9	99.0	189	39	45.1	3.9	0.8	0.0	0.82	0.01	nd	nd
67	35	4.48	0.61	3.62	0.23	0.0	0.0	4.58	12.0	37.7	398	82.3	26.2	31.8	1.3	1.94	98.9	156	36	45.8	5.1	1.0	0.1	0.84	0.03	nd	nd
71	39	4.04	0.57	3.25	0.20	0.0	0.0	4.70	12.4	37.9	452	80.6	26.4	32.7	1.7	1.96	98.7	150	33	41.2	5.2	1.2	0.0	0.91	0.11	nd	nd
74	42	4.14	1.09	2.87	0.17	0.0	0.0	4.73	12.5	38.1	440	80.5	26.4	32.8	1.2	1.96	98.6	173	42	nd	nd	nd	nd	nd	nd	nd	nd

Baboon T2

Age	Challenge Day	WBC Total	WBC Neut	WBC Lymph	WBC Mono	WBC Eosin	WBC Baso	RBC	HGB	HCT	PLT	MCV	MCH	MCHC	Retic	Weight	Avg Temp	Avg HR	Avg Resp	serum anti-FHA		serum anti-PTx		serum ADA		NPW anti-PTx	
days	days	x10^3/µl	x10^3/µl	x10^3/µl	x10^3/µl	x10^3/µl	x10^3/µl	x10^6/µl	g/dL	%	x10^3/µl	fL	pg	%	%	kg	°F	/min	/min	IU/ml	+/-	µg/ml	+/-	rel abs	+/-	ng/ml	+/-
1	-39	13.80	10.8	1.71	1.16	0.0	0.1	5.25	16.3	51.0	190	97.1	31.0	32.0	4.2	1.0	97.8	161	61	0.5	0.0	0.1	0.1	0.11	0.00	nd	nd
7	-33	5.81	2.35	2.77	0.65	0.0	0.0	5.68	16.8	52.5	243	92.4	29.6	32.0	0.6	nd	nd	nd	nd	0.7	0.1	207.6	18.6	0.11	0.01	nd	nd
14	-26	9.46	3.14	5.33	0.91	0.0	0.0	5.17	15.0	45.7	246	88.4	29.0	32.8	3.0	nd	nd	nd	nd	0.7	0.0	86.4	18.8	0.19	0.01	nd	nd
21	-19	7.40	1.29	5.30	0.69	0.1	0.1	5.26	14.8	45.0	179	85.6	28.1	32.9	2.9	nd	nd	nd	nd	0.7	0.0	54.1	6.0	0.35	0.00	nd	nd
28	-12	7.25	2.40	4.30	0.49	0.1	0.0	4.72	12.8	40.6	202	86.0	27.1	31.5	5.2	nd	nd	nd	nd	0.6	0.0	16.0	2.4	0.57	0.01	nd	nd
35	-5	10.53	4.03	6.00	0.42	0.1	0.0	5.01	13.6	42.3	244	84.4	27.1	32.2	4.7	nd	nd	nd	nd	0.7	0.0	0.4	0.1	0.80	0.00	nd	nd
40	0	7.70	5.53	1.96	0.18	0.0	0.0	4.80	12.8	39.7	247	82.7	26.7	32.2	3.7	nd	99.0	nd	nd	0.9	0.2	0.1	0.0	0.81	0.01	nd	nd
43	3	17.49	2.36	14.3	0.58	0.2	0.0	5.03	13.0	41.5	215	82.5	25.8	31.3	3.0	1.6	98.7	200	62	0.5	0.0	0.1	0.0	0.84	0.01	0.1	0.0
47	7	59.18	17.2	34.6	6.92	0.5	0.0	4.83	12.5	40.8	325	84.5	25.9	30.6	1.6	1.54	100.6	248	99	0.5	0.0	0.1	0.0	0.86	0.01	nd	nd
50	10	75.51	26.4	39.1	9.59	0.4	0.1	4.87	12.3	40.3	240	82.8	25.3	30.5	1.4	nd	99.4	256	110	0.3	0.0	0.0	0.0	0.86	0.01	nd	nd
54	14	64.05	26.1	30.7	6.85	0.3	0.1	4.79	11.8	38.4	105	80.2	24.6	30.7	2.2	nd	98.2	230	149	4.7	0.0	0.0	0.0	0.79	0.02	nd	nd

Baboon T3

Age	Challenge Day	WBC Total	WBC Neut	WBC Lymph	WBC Mono	WBC Eosin	WBC Baso	RBC	HGB	HCT	PLT	MCV	MCH	MCHC	Retic	Weight	Avg Temp	Avg HR	Avg Resp	serum anti-FHA		serum anti-PTx		serum ADA		NPW anti-PTx	
days	days	x10^3/µl	x10^3/µl	x10^3/µl	x10^3/µl	x10^3/µl	x10^3/µl	x10^6/µl	g/dL	%	x10^3/µl	fL	pg	%	%	kg	°F	/min	/min	IU/ml	+/-	µg/ml	+/-	rel abs	+/-	ng/ml	+/-
1	-33	11.60	7.69	2.90	0.93	0.0	0.0	6.06	18.4	57.2	230	94.4	30.4	32.2	2.1	1.1	97.6	153	47	8.4	0.4	0.07	0.02	0.11	0.00	nd	nd
8	-26	5.42	1.82	2.79	0.78	0.0	0.0	5.80	17.0	52.2	235	90.0	29.3	32.6	0.5	nd	nd	nd	nd	4.9	0.1	171.9	15.7	0.11	0.01	nd	nd
15	-19	7.20	3.20	3.29	0.66	0.0	0.0	5.37	15.2	47.2	162	87.9	28.3	32.2	1.2	nd	nd	nd	nd	7.2	0.3	78.0	8.8	0.12	0.00	nd	nd
22	-12	8.01	3.17	4.17	0.65	0.0	0.0	5.03	13.9	42.7	246	84.9	27.6	32.6	1.4	nd	nd	nd	nd	5.3	0.2	85.9	7.7	0.13	0.00	nd	nd
29	-5	7.11	3.21	3.50	0.40	0.0	0.0	4.91	12.9	40.9	323	83.3	26.3	31.5	2.1	nd	nd	nd	nd	5.3	0.1	51.6	0.2	0.20	0.01	nd	nd
34	0	5.62	3.80	1.58	0.23	0.0	0.0	4.73	12.2	38.0	279	80.3	25.8	32.1	2.4	nd	98.5	181	46	4.1	0.5	30.1	0.9	0.25	0.00	nd	nd
37	3	5.63	3.03	2.19	0.40	0.0	0.0	4.77	12.2	37.8	256	79.2	25.6	32.3	2.5	1.51	97.7	163	85	2.9	0.1	18.4	0.5	0.30	0.01	2.9	0.1
41	7	5.10	1.72	2.98	0.38	0.0	0.0	4.79	12.1	37.8	306	78.9	25.3	32.0	2.4	1.55	98.1	177	63	2.4	0.1	4.5	0.1	0.32	0.02	nd	nd
44	10	8.56	3.28	4.67	0.57	0.0	0.0	4.82	12.1	37.8	328	78.4	25.1	32.0	2.5	1.60	97.9	153	41	2.2	0.0	2.5	0.0	0.36	0.01	nd	nd
48	14	13.16	7.66	4.94	0.53	0.0	0.0	5.09	12.6	39.8	340	78.2	24.8	31.7	2.3	1.66	96.7	148	42	5.2	0.3	1.3	0.2	0.43	0.00	nd	nd
51	17	9.05	3.59	4.95	0.41	0.0	0.1	5.20	12.9	40.5	269	77.9	24.8	31.9	1.9	1.67	97.2	135	32	13.3	1.0	2.0	0.2	0.43	0.01	nd	nd
55	21	7.74	3.92	3.44	0.37	0.0	0.0	5.17	12.7	39.8	364	77.0	24.6	31.9	1.1	1.72	98.0	163	52	69.8	4.6	1.1	0.1	0.46	0.00	nd	nd
58	24	5.72	2.09	3.29	0.28	0.1	0.0	4.98	12.1	37.8	340	75.9	24.3	32.0	1.0	1.77	98.8	184	51	93.2	6.1	1.1	0.0	0.45	0.02	nd	nd
62	28	4.63	2.62	1.81	0.18	0.0	0.0	5.46	13.4	40.7	314	74.5	24.5	32.9	0.8	1.70	98.2	130	39	107.6	14.6	1.7	0.1	0.46	0.02	nd	nd
65	31	3.91	1.63	2.05	0.22	0.0	0.0	5.13	12.5	38.2	255	74.5	24.4	32.7	1.0	nd	97.7	161	41	186.7	0.4	1.8	0.1	0.44	0.00	nd	nd
69	35	4.36	2.01	2.16	0.18	0.0	0.0	5.21	12.5	39.2	311	75.2	24.0	31.9	1.6	1.85	98.0	134	51	238.4	8.1	1.6	0.0	0.40	0.00	nd	nd
72	38	4.05	1.66	2.19	0.18	0.0	0.0	5.19	12.5	38.9	214	75.0	24.1	32.1	1.2	1.91	98.8	129	48	293.9	32.2	1.6	0.1	0.43	0.00	nd	nd
76	42	nd	nd	nd	nd	nd	nd	nd	nd	nd	nd	nd	nd	nd	nd	nd	nd	nd	nd	304.4	39.4	1.5	0.2	0.42	0.01	nd	nd

Baboon T4

Age	Challenge Day	WBC															Avg	Avg	Avg	serum		serum		serum		NPW	
Age	Day	Total	Neut	Lymph	Mono	Eosin	Baso	RBC	HGB	HCT	PLT	MCV	MCH	MCHC	Retic	Weight	Temp	HR	Resp	anti-FHA		anti-PTx		ADA		anti-PTx	
days	days	$x10^3$/µl	$x10^3$/µl	$x10^3$/µl	$x10^3$/µl	$x10^3$/µl	$x10^3$/µl	$x10^6$/µl	g/dL	%	$x10^3$/µl	fL	pg	%	%	kg	°F	/min	/min	IU/ml	+/-	µg/ml	+/-	rel abs	+/-	ng/ml	+/-
1	-31	7.63	5.04	1.95	0.60	0.0	0.0	5.14	16.4	49.3	416	95.9	31.9	33.3	4.0	1.0	97.9	162	82	10.6	1.3	0.19	0.01	0.10	0.00	nd	nd
8	-24	nd	nd	nd	nd	nd	nd	nd	nd	nd	nd	nd	nd	nd	nd	nd	nd	nd	nd	7.9	1.5	157.9	53.8	0.10	0.00	nd	nd
14	-18	5.65	1.95	3.09	0.54	0.1	0.0	4.59	13.4	37.2	559	81.0	29.2	36.0	1.9	nd	nd	nd	nd	7.1	1.0	52.3	5.6	0.10	0.01	nd	nd
22	-10	6.04	2.00	3.65	0.32	0.1	0.0	4.25	11.8	33.4	461	78.6	27.8	35.3	3.6	nd	nd	nd	nd	6.1	0.2	36.6	5.8	0.11	0.00	nd	nd
28	-4	5.25	1.14	3.52	0.53	0.1	0.0	4.12	10.9	31.7	374	76.9	26.5	34.4	2.2	nd	nd	nd	nd	5.6	0.3	23.1	8.6	0.12	0.00	nd	nd
32	0	4.95	1.23	3.30	0.37	0.0	0.0	4.21	10.9	33.0	512	78.4	25.9	33.0	4.3	1.2	98.5	172.3	59	3.8	0.6	37.0	9.6	0.14	0.01	nd	nd
36	4	12.91	1.45	10.6	0.68	0.2	0.0	4.09	10.3	31.1	556	76.0	25.2	33.1	3.1	1.3	99.6	141	93	4.4	0.2	36.6	4.9	0.16	0.01	2.0	0.4
39	7	20.07	2.45	16.6	0.84	0.2	0.0	4.07	10.1	31.3	538	76.9	24.8	32.3	4.6	1.48	99.5	188	81	4.9	0.5	29.1	4.6	0.18	0.02	nd	nd
43	11	23.46	4.48	17.9	0.87	0.1	0.0	4.25	10.4	32.7	427	76.9	24.5	31.8	6.2	1.50	98.4	nd	nd	8.5	1.1	23.4	2.1	0.18	0.01	nd	nd
46	14	20.04	3.21	15.9	0.78	0.1	0.0	4.59	11.0	34.6	524	75.4	24.0	31.8	4.6	1.50	99.2	180	48	26.8	2.5	22.2	1.3	0.25	0.00	nd	nd
50	18	12.60	3.65	8.40	0.48	0.1	0.0	4.75	11.4	35.4	526	74.5	24.0	32.2	2.6	1.53	99.0	189	86	61.3	4.6	10.9	1.4	0.26	0.01	nd	nd
53	21	7.64	2.00	5.12	0.47	0.0	0.0	4.54	10.8	34.7	475	76.4	23.8	31.1	2.5	1.60	98.5	146	75	76.5	3.1	11.8	0.0	0.25	0.00	nd	nd
57	25	4.93	1.50	3.04	0.34	0.0	0.0	4.80	11.5	34.7	592	72.3	24.0	33.1	2.2	1.61	98.9	163	48	99.8	4.8	10.1	1.0	0.25	0.01	nd	nd
60	28	5.14	1.98	2.86	0.27	0.0	0.0	4.66	11.0	34.4	529	73.8	23.6	32.0	1.8	1.67	99.7	176	48	92.1	4.9	19.4	1.6	0.26	0.01	nd	nd
64	32	4.59	1.19	3.04	0.33	0.0	0.0	4.76	11.2	35.2	527	73.9	23.5	31.8	2.7	1.70	99.1	168	57	113.9	9.5	13.1	0.1	0.27	0.01	nd	nd
67	35	4.18	1.30	2.65	0.21	0.0	0.0	4.89	11.5	36.4	510	74.4	23.5	31.6	2.7	1.77	98.5	160	56	109.8	5.0	12.3	0.2	0.29	0.02	nd	nd
71	39	5.65	2.44	2.95	0.24	0.0	0.0	4.75	11.1	35.2	407	74.1	23.4	31.5	2.3	1.88	98.9	143	57	108.7	1.4	11.0	0.1	0.28	0.00	nd	nd
74	42	4.38	0.95	3.23	0.16	0.0	0.0	5.02	11.8	36.8	428	73.3	23.5	32.1	2.2	nd	nd	nd	nd	98.1	0.6	10.5	0.1	0.31	0.01	nd	nd

Baboon T5

Age	Challenge Day	WBC Total	WBC Neut	WBC Lymph	WBC Mono	WBC Eosin	WBC Baso	RBC	HGB	HCT	PLT	MCV	MCH	MCHC	Retic	Weight	Avg Temp	Avg HR	Avg Resp	serum anti-FHA	+/-	serum anti-PTx	+/-	serum ADA	+/-	NPW anti-PTx	+/-
days	days	x10³/µl	x10³/µl	x10³/µl	x10³/µl	x10³/µl	x10³/µl	x10⁶/µl	g/dL	%	x10³/µl	fL	pg	%	%	kg	°F	/min	/min	IU/ml	+/-	µg/ml	+/-	rel abs	+/-	ng/ml	+/-
1	-33	6.75	3.79	2.24	0.64	0.1	0.0	5.35	16.0	49.3	296	92.1	29.9	32.5	2.0	nd	98.9	148	40	0.3	0.1	0.03	0.01	0.12	0.00	nd	nd
9	-25	6.1	1.26	3.75	1.01	0.1	0.0	5.84	16.6	48.9	217	83.7	28.4	33.9	0.34	nd	nd	nd	nd	0.5	0.0	172.9	35.0	0.10	0.00	nd	nd
16	-18	6.81	1.97	3.87	0.87	0.0	0.1	5.08	14.1	44.1	442	86.8	27.8	32.0	3.4	nd	nd	nd	nd	0.6	0.0	99.1	15.2	0.10	0.00	nd	nd
25	-9	8.66	2.80	4.78	0.99	0.1	0.0	5.18	14.2	44.1	226	85.1	27.4	32.2	3.2	nd	nd	nd	nd	0.4	0.0	66.2	6.0	0.11	0.00	nd	nd
27	-7	7.55	2.15	4.52	0.78	0.1	0.0	4.68	12.7	40.0	464	85.5	27.1	31.8	3.7	nd	nd	nd	nd	0.4	0.0	45.4	4.5	0.10	0.00	nd	nd
34	0	6.90	3.42	3.11	0.32	0.0	0.0	5.12	13.4	42.1	555	82.2	26.2	31.8	3.0	1.35	99.4	165.5	38.0	0.5	0.0	32.0	3.1	0.11	0.00	nd	nd
38	4	7.25	2.44	3.98	0.75	0.1	0.0	5.00	13.0	40.1	378	80.2	26.0	32.4	1.3	1.34	98.0	158	32	1.3	0.1	24.4	1.1	0.11	0.00	0.8	0.1
41	7	7.51	2.35	4.29	0.78	0.1	0.0	4.75	12.3	37.9	464	79.8	25.9	32.5	2.4	1.38	98.9	157	36	1.5	0.1	15.0	1.2	0.11	0.00	nd	nd
45	11	11.48	3.17	7.76	0.51	0.0	0.0	4.87	12.5	39.0	491	80.1	25.7	32.1	3.3	1.42	98.9	152	42	2.4	0.1	14.7	0.6	0.11	0.00	nd	nd
48	14	12.74	3.57	8.61	0.51	0.0	0.0	4.84	12.2	38.9	433	80.4	25.2	31.4	3.3	1.51	98.9	156	66	4.6	0.1	10.8	0.2	0.10	0.00	nd	nd
52	18	15.30	8.48	6.46	0.32	0.0	0.0	4.65	11.9	37.4	440	80.4	25.6	31.8	2.9	1.67	97.8	184	54	6.8	0.4	10.2	0.7	0.12	0.01	nd	nd
55	21	7.39	1.66	5.20	0.50	0.0	0.0	4.80	12.3	38.7	442	80.6	25.6	31.8	3.2	1.64	99.1	151	42	4.7	0.2	17.6	0.2	0.13	0.01	nd	nd
59	25	7.03	2.09	4.52	0.39	0.0	0.0	5.01	12.8	40.6	513	81.0	25.5	31.5	2.5	1.66	98.3	155	32	10.9	0.4	18.1	2.4	0.13	0.00	nd	nd
62	28	5.95	1.14	4.46	0.32	0.0	0.0	4.98	12.6	39.8	462	79.9	25.3	31.7	1.9	1.72	100.0	190	41	20.2	3.0	16.2	0.5	0.13	0.00	nd	nd
66	32	5.05	0.87	3.67	0.42	0.1	0.0	5.09	12.8	40.3	482	79.2	25.1	31.8	1.9	1.69	98.6	158	36	35.3	3.4	13.9	0.2	0.12	0.00	nd	nd
69	35	5.32	1.95	3.05	0.28	0.0	0.0	4.91	12.4	38.5	471	78.4	25.3	32.2	2.4	1.74	99.1	167	32	48.5	4.3	11.9	1.2	0.12	0.00	nd	nd
73	39	5.02	1.01	3.69	0.26	0.1	0.0	5.03	12.6	39.5	461	78.5	25.0	31.9	2.2	1.81	99.1	158	31	69.2	4.4	13.0	0.2	0.12	0.00	nd	nd
76	42	7.32	3.00	3.89	0.38	0.1	0.0	5.22	12.9	40.8	403	78.2	24.7	31.6	2.4	1.84	99.3	161	30	79.0	8.3	11.3	2.9	0.12	0.00	nd	nd

Baboon T6

Age	Challenge Day	WBC Total	Neut	Lymph	Mono	Eosin	Baso	RBC	HGB	HCT	PLT	MCV	MCH	MCHC	Retic	Weight	Avg Temp	Avg HR	Avg Resp	serum anti-FHA		serum anti-PTx		serum ADA		NPW anti-PTx	
days	days	x10³/µl	x10³/µl	x10³/µl	x10³/µl	x10³/µl	x10³/µl	x10⁶/µl	g/dL	%	x10³/µl	fL	pg	%	%	kg	°F	/min	/min	IU/ml	+/-	µg/ml	+/-	rel abs	+/-	ng/ml	+/-
1	-27	9.30	8.03	0.93	0.33	0.0	0.0	4.22	12.5	36.5	369	86.5	29.6	34.2	2.0	nd	nd	nd	nd	0.8	0.1	0.07	0.02	0.10	0.00	nd	nd
7	-21	4.94	1.69	2.51	0.67	0.1	0.0	4.39	12.2	37.6	346	85.6	27.8	32.4	0.3	nd	nd	nd	nd	1.0	0.2	200.2	35.1	0.10	0.00	nd	nd
14	-14	4.84	2.06	2.17	0.51	0.1	0.0	4.06	11.0	35.2	457	86.7	27.1	31.3	4.3	nd	nd	nd	nd	0.7	0.0	90.4	10.3	0.10	0.01	nd	nd
21	-7	5.56	2.27	2.68	0.47	0.1	0.0	3.98	10.2	33.4	379	83.9	25.6	30.5	5.2	nd	nd	nd	nd	0.8	0.0	69.9	21.4	0.11	0.00	nd	nd
28	0	4.93	2.97	1.67	0.26	0.0	0.0	4.44	11.1	35.9	443	80.9	25.0	30.9	3.8	1.10	98.3	158	72	0.8	0.0	45.6	7.5	0.12	0.01	nd	nd
32	4	3.39	1.13	1.96	0.23	0.1	0.0	4.17	10.4	33.1	352	79.4	24.9	31.4	1.7	1.10	96.9	150	54	1.0	0.0	47.2	13.0	0.14	0.01	2.7	0.2
35	7	5.63	1.71	3.57	0.28	0.1	0.0	4.48	11.0	35.3	423	78.8	24.6	31.2	2.2	1.11	99.8	140	90	1.2	0.0	44.6	10.5	0.17	0.01	nd	nd
39	11	9.14	2.19	6.43	0.39	0.1	0.0	4.44	10.8	35.9	441	80.9	24.3	30.1	2.3	1.19	98.8	140	68	1.7	0.1	33.0	3.7	0.20	0.04	nd	nd
42	14	11.30	3.23	7.59	0.38	0.1	0.0	4.45	10.9	35.9	412	80.7	24.5	30.4	3.4	1.23	98.0	146	62	14.6	0.7	26.5	0.6	0.24	0.04	nd	nd
46	18	10.80	5.58	4.65	0.51	0.0	0.0	4.49	11.0	35.9	336	80.0	24.5	30.6	2.5	1.31	98.1	138	50	19.9	1.7	18.9	5.3	0.34	0.02	nd	nd
49	21	5.57	1.94	3.28	0.30	0.0	0.0	4.60	11.2	36.6	317	79.6	24.3	30.6	2.4	1.35	98.5	126	66	34.1	3.4	12.5	0.1	0.43	0.01	nd	nd
53	25	4.83	1.56	2.91	0.30	0.0	0.0	4.83	11.7	38.7	381	80.1	24.2	30.2	2.6	1.41	97.8	131	60	63.8	2.8	9.2	0.0	0.60	0.00	nd	nd
56	28	4.40	1.98	2.20	0.20	0.0	0.0	5.03	12.2	39.7	262	78.9	24.3	30.7	2.0	1.45	99.7	173	46	99.6	0.7	7.6	0.5	0.65	0.05	nd	nd
60	32	3.26	1.01	2.03	0.18	0.0	0.0	4.98	12.1	38.8	386	77.9	24.3	31.2	1.3	1.44	98.5	151	59	151.5	10.4	14.3	3.4	0.74	0.03	nd	nd
63	35	3.84	1.84	1.81	0.17	0.0	0.0	5.05	12.2	39.2	347	77.6	24.2	31.1	1.6	nd	98.9	152	48	149.0	32.0	13.0	0.8	0.82	0.04	nd	nd
67	39	3.70	1.39	2.00	0.22	0.1	0.0	4.95	11.9	38.8	340	78.4	24.0	30.7	1.1	nd	99.6	141	58	215.9	27.1	11.8	0.6	0.90	0.02	nd	nd
70	42	3.69	1.73	1.76	0.18	0.0	0.0	4.94	11.8	38.1	341	77.1	23.9	31.0	1.4	1.58	100.1	188	60	193.1	19.1	11.9	0.8	0.94	0.03	nd	nd

Baboon T7

Age	Challenge Day	WBC Total	WBC Neut	WBC Lymph	WBC Mono	WBC Eosin	WBC Baso	RBC	HGB	HCT	PLT	MCV	MCH	MCHC	Retic	Weight	Avg Temp	Avg HR	Avg Resp	serum anti-FHA		serum anti-PTx		serum ADA		NPW anti-PTx	
days	days	$x10^3$/µl	$x10^3$/µl	$x10^3$/µl	$x10^3$/µl	$x10^3$/µl	$x10^3$/µl	$x10^6$/µl	g/dL	%	$x10^3$/µl	fL	pg	%	%	kg	°F	/min	/min	IU/ml	+/-	µg/ml	+/-	rel abs	+/-	ng/ml	+/-
1	-28	8.16	6.28	1.26	0.61	0.0	0.0	4.98	15.1	46.4	480	93.2	30.3	32.5	1.5	0.76	97.0	136.7	51.3	0.4	0.0	0.07	0.01	0.11	0.00	nd	nd
8	-21	4.99	0.93	3.08	0.91	0.1	0.0	4.97	14.6	45.3	416	91.1	29.4	32.2	0.4	nd	nd	nd	nd	0.5	0.0	138.2	15.4	0.12	0.00	nd	nd
15	-14	6.43	1.56	4.33	0.42	0.1	0.0	4.35	12.5	38.8	568	89.2	28.7	32.2	2.4	nd	nd	nd	nd	0.5	0.0	96.6	10.2	0.11	0.00	nd	nd
22	-7	7.03	1.60	4.69	0.53	0.2	0.0	4.45	12.4	39.2	476	88.1	27.9	31.6	2.5	nd	nd	nd	nd	0.6	0.0	64.6	13.1	0.13	0.01	nd	nd
29	0	5.82	1.46	3.87	0.42	0.1	0.0	4.25	11.5	37.0	472	87.1	27.1	31.1	4.2	1.08	98.8	160.0	51.0	0.6	0.0	59.5	11.5	0.20	0.01	nd	nd
31	2	2.87	1.11	1.61	0.14	0.0	0.0	3.99	10.7	31.8	400	79.7	26.8	33.6	3.0	1.03	98.3	136.0	53.0	1.7	0.0	55.7	2.8	0.22	0.02	0.7	0.1
35	6	4.61	0.78	3.39	0.38	0.1	0.0	4.06	10.7	33.2	355	81.8	26.4	32.2	1.9	1.03	98.1	140	39	1.8	0.2	54.9	3.1	0.25	0.01	nd	nd
38	9	8.02	1.12	6.39	0.42	0.1	0.0	4.09	10.6	33.5	409	81.9	25.9	31.6	3.4	1.10	98.2	144	46	2.3	0.4	41.6	7.3	0.28	0.02	nd	nd
42	13	12.48	2.06	9.71	0.59	0.1	0.0	4.05	10.5	33.6	463	83.0	25.9	31.3	4.0	1.19	98.4	181	54	6.7	0.7	40.0	9.5	0.34	0.00	nd	nd
45	16	9.73	1.22	8.04	0.38	0.1	0.0	4.13	10.7	34.1	317	82.6	25.9	31.4	4.0	1.23	98.7	141	48	9.7	1.5	30.8	6.3	0.37	0.01	nd	nd
49	20	6.72	1.01	5.17	0.49	0.0	0.0	4.42	11.3	36.3	461	82.1	25.6	31.1	3.6	1.28	98.2	142	40	13.9	0.8	19.5	0.7	0.54	0.01	nd	nd
52	23	5.13	0.62	4.12	0.34	0.1	0.0	4.36	11.2	35.0	491	80.3	25.7	32.0	2.2	1.30	99.6	183	45	26.4	0.4	9.1	1.9	0.63	0.05	nd	nd
56	27	5.19	1.16	3.45	0.52	0.1	0.0	4.54	11.5	36.2	513	79.7	25.3	31.8	2.0	1.32	98.4	190	52	74.4	0.8	6.1	0.2	0.77	0.00	nd	nd
59	30	4.65	1.45	2.99	0.17	0.0	0.0	4.35	11.1	34.8	515	80.0	25.5	31.9	2.0	nd	99.3	178	44	86.0	11.4	7.0	0.5	0.85	0.02	nd	nd
63	34	4.77	1.41	3.09	0.25	0.0	0.0	4.72	11.9	37.6	519	79.7	25.2	31.6	2.8	nd	99.7	186	52	110.6	4.7	5.2	0.6	0.96	0.01	nd	nd
66	37	4.78	1.26	3.27	0.23	0.0	0.0	4.61	11.5	36.3	488	78.7	24.9	31.7	2.0	nd	97.9	153	45	83.9	1.0	3.5	2.2	0.97	0.01	nd	nd
70	41	4.17	0.91	2.93	0.29	0.0	0.0	4.40	11.1	34.8	476	79.1	25.2	31.9	2.4	nd	99.8	178	51	132.7	2.9	6.5	0.5	1.04	0.01	nd	nd
71	42	5.56	2.19	2.96	0.40	0.0	0.0	4.82	12.1	37.9	519	78.6	25.1	31.9	3.1	1.54	nd	nd	nd	nd	nd	nd	nd	nd	nd	nd	nd

Baboon T8

		WBC																		serum		serum		serum		NPW	
Age	Challenge Day	Total	Neut	Lymph	Mono	Eosin	Baso	RBC	HGB	HCT	PLT	MCV	MCH	MCHC	Retic	Weight	Avg Temp	Avg HR	Avg Resp	anti-FHA		anti-PTx		ADA		anti-PTx	
days	days	x10^3/µl	x10^3/µl	x10^3/µl	x10^3/µl	x10^3/µl	x10^3/µl	x10^6/µl	g/dL	%	x10^3/µl	fL	pg	%	%	kg	°F	/min	/min	IU/ml	+/-	µg/ml	+/-	rel abs	+/-	ng/ml	+/-
1	-28	9.43	7.91	0.84	0.64	0.0	0.0	5.57	16.9	52.9	225	95.0	30.3	31.9	3.7	1.36	99.3	170	83	4.5	0.4	0.04	0.00	0.11	0.01	nd	nd
8	-21	7.98	4.55	2.63	0.73	0.0	0.0	5.89	16.8	51.1	397	86.8	28.5	32.9	0.64	nd	nd	nd	nd	5.0	0.0	181.2	20.3	0.11	0.01	nd	nd
15	-14	6.61	3.46	2.57	0.53	0.0	0.0	5.70	15.2	46.2	425	81.1	26.7	32.9	1.0	nd	nd	nd	nd	3.7	0.2	82.9	3.4	0.11	0.00	nd	nd
22	-7	8.31	4.78	2.96	0.52	0.0	0.0	5.85	15.2	46.2	297	79.0	26.0	32.9	1.9	nd	nd	nd	nd	2.5	0.4	62.8	10.0	0.10	0.00	nd	nd
29	0	10.47	7.59	2.46	0.32	0.0	0.1	5.17	13.4	41.0	313	79.3	25.9	32.7	1.6	1.61	98.6	182	63	3.5	0.1	53.0	22.1	0.11	0.00	nd	nd
31	2	5.44	3.43	1.70	0.23	0.1	0.0	5.24	13.3	40.2	290	76.7	25.4	33.1	1.3	1.59	97.7	149	63	4.1	0.0	46.0	4.7	0.11	0.00	0.7	0.0
35	6	6.24	1.94	3.99	0.20	0.1	0.0	4.92	12.4	35.9	373	73.0	25.2	34.5	1.0	1.62	98.7	187	51	5.1	0.3	23.8	1.8	0.12	0.01	nd	nd
38	9	10.57	4.11	6.07	0.30	0.1	0.0	4.80	11.8	36.0	380	75.0	24.6	32.8	1.7	1.64	97.9	142	54	5.8	0.0	21.0	3.4	0.12	0.01	nd	nd
42	13	18.05	9.82	7.83	0.32	0.1	0.0	4.76	11.7	36.5	312	76.7	24.6	32.1	2.5	1.75	97.6	158	50	10.1	0.5	18.4	1.3	0.12	0.01	nd	nd
45	16	12.07	3.40	8.20	0.37	0.1	0.0	4.86	11.8	36.6	334	75.3	24.3	32.2	2.6	1.80	98.1	159	51	23.3	2.2	15.1	0.3	0.12	0.00	nd	nd
49	20	9.92	2.59	7.00	0.26	0.1	0.0	5.26	12.7	39.9	412	75.9	24.1	31.8	2.1	1.83	98.6	144	64	36.6	2.8	20.5	0.5	0.13	0.00	nd	nd
52	23	6.49	1.88	4.41	0.15	0.0	0.0	4.93	11.8	36.9	377	74.8	23.9	32.0	1.3	1.88	99.1	153	48	45.3	2.1	15.5	0.8	0.13	0.01	nd	nd
56	27	4.54	1.50	2.82	0.18	0.0	0.0	5.06	12.0	37.5	361	74.1	23.7	32.0	1.5	1.87	99.0	136	60	80.1	4.4	13.6	0.4	0.13	0.00	nd	nd
59	30	6.62	4.52	1.90	0.17	0.0	0.0	5.00	11.9	37.1	370	74.2	23.8	32.1	1.8	nd	97.8	143	46	68.6	10.5	10.0	0.1	0.13	0.00	nd	nd
63	34	3.91	1.70	2.01	0.16	0.0	0.0	4.88	11.5	35.8	333	73.4	23.6	32.1	1.5	nd	99.2	157	48	151.0	19.4	8.5	1.5	0.12	0.00	nd	nd
66	37	5.52	3.32	1.97	0.22	0.0	0.0	4.76	11.4	35.0	305	73.5	23.9	32.6	2.4	nd	97.7	167	48	147.4	3.8	23.7	0.7	0.12	0.00	nd	nd
70	41	4.69	2.53	1.96	0.16	0.0	0.0	4.86	11.4	35.9	300	73.9	23.5	31.8	2.1	nd	99.4	188	60	133.2	8.4	22.0	4.6	0.13	0.01	nd	nd
71	42	9.41	6.90	2.18	0.31	0.0	0.0	5.05	11.9	37.4	279	74.1	23.6	31.8	2.5	2.13	nd	nd	nd	nd	nd	nd	nd	nd	nd	nd	nd

Baboon Y1

Age	Challenge Day	WBC Total	WBC Neut	WBC Lymph	WBC Mono	WBC Eosin	WBC Baso	RBC	HGB	HCT	PLT	MCV	MCH	MCHC	Retic	Weight	Avg Temp	Avg HR	Avg Resp	serum anti-FHA		serum anti-PTx		serum ADA		NPW anti-PTx	
days	days	x10³/µl	x10³/µl	x10³/µl	x10³/µl	x10³/µl	x10³/µl	x10⁶/µl	g/dL	%	x10³/µl	fL	pg	%	%	kg	°F	/min	/min	IU/ml	+/-	µg/ml	+/-	rel abs	+/-	ng/ml	+/-
1	-33	nd	nd	nd	nd	nd	nd	nd	nd	nd	nd	nd	nd	nd	nd	0.90	nd	nd	nd	1.1	0.0	0.64	0.00	0.13	0.01	nd	nd
7	-27	nd	nd	nd	nd	nd	nd	nd	nd	nd	nd	nd	nd	nd	nd	nd	nd	nd	nd	1.2	0.2	174.3	26.5	0.14	0.00	nd	nd
14	-20	7.76	2.44	4.60	0.65	0.1	0.0	4.69	14.4	43.0	424	91.7	30.7	33.5	0.9	nd	nd	nd	nd	1.0	0.0	191.6	6.0	0.13	0.00	nd	nd
21	-13	8.89	3.21	4.88	0.74	0.1	0.0	4.42	13.2	40.1	381	90.7	29.9	32.9	2.9	nd	nd	nd	nd	1.0	0.1	146.5	19.3	0.09	0.00	nd	nd
28	-6	7.08	2.61	3.94	0.49	0.0	0.0	4.82	14.0	43.0	431	89.2	29.0	32.6	3.2	nd	nd	nd	nd	1.0	0.0	139.6	14.1	0.13	0.00	nd	nd
34	0	5.37	1.87	3.23	0.25	0.0	0.0	4.60	12.8	40.1	430	87.2	27.8	31.9	1.7	1.15	98.9	166.3	38.0	0.4	0.0	66.7	10.1	0.14	0.00	nd	nd
37	3	7.04	2.26	4.01	0.65	0.1	0.0	4.11	11.3	35.2	345	85.6	27.5	32.1	2.0	1.25	98.9	210	64	0.3	0.0	48.4	7.6	0.12	0.01	8.3	0.7
41	7	10.27	4.72	5.15	0.37	0.0	0.0	4.19	11.4	35.1	497	83.8	27.2	32.5	4.8	nd	98.4	189	58	0.2	0.0	36.0	8.9	0.13	0.01	nd	nd
44	10	11.37	4.20	6.55	0.56	0.1	0.0	4.41	11.8	37.6	575	85.3	26.8	31.4	4.2	1.29	98.5	186	38	0.9	0.1	38.7	2.3	0.13	0.01	nd	nd
48	14	13.41	3.93	8.72	0.67	0.1	0.0	4.44	11.7	37.4	490	84.2	26.4	31.3	2.6	1.36	99.4	194	38	1.4	0.1	78.4	29.4	0.13	0.00	nd	nd
51	17	10.31	3.81	5.86	0.57	0.1	0.0	4.73	12.3	39.1	482	82.7	26.0	31.5	3.1	1.40	99.4	188	56	3.2	0.4	38.7	7.4	0.13	0.00	nd	nd
55	21	9.47	3.31	5.39	0.69	0.1	0.0	4.43	11.6	36.1	418	81.5	26.2	32.1	3.3	1.51	99.1	199	48	16.7	0.5	29.8	4.5	0.14	0.00	nd	nd
58	24	6.49	2.35	3.76	0.37	0.0	0.0	4.70	12.2	38.9	418	82.8	26.0	31.4	2.6	1.46	99.5	182	44	32.9	3.4	27.1	0.6	0.13	0.01	nd	nd
62	28	9.01	3.69	4.85	0.44	0.0	0.0	4.71	11.9	38.6	455	82.0	25.3	30.8	2.1	1.55	98.6	181	48	38.7	1.7	23.8	1.6	0.13	0.00	nd	nd
65	31	7.90	2.98	4.56	0.30	0.1	0.0	4.63	11.8	38.3	485	82.7	25.5	30.8	2.8	1.61	98.5	180	53	39.4	6.2	41.0	9.0	0.13	0.00	nd	nd
69	35	7.83	2.57	4.85	0.32	0.1	0.0	4.67	11.8	38.5	734	82.4	25.3	30.6	2.5	1.66	99.6	166	39	51.1	0.8	26.9	4.7	0.09	0.00	nd	nd
72	38	9.57	3.72	5.38	0.39	0.1	0.0	4.63	12.0	38.2	454	82.5	25.9	31.4	3.5	1.75	nd	nd	nd	53.5	8.7	18.4	0.1	0.12	0.00	nd	nd
76	42	7.13	2.08	4.56	0.39	0.1	0.0	4.77	12.2	39.2	492	82.2	25.6	31.1	2.8	1.77	100.1	215	48	55.0	0.5	19.1	0.3	0.12	0.00	nd	nd

Baboon Y2

Age days	Challenge Day days	WBC Total $x10^3/\mu l$	WBC Neut $x10^3/\mu l$	WBC Lymph $x10^3/\mu l$	WBC Mono $x10^3/\mu l$	WBC Eosin $x10^3/\mu l$	WBC Baso $x10^3/\mu l$	RBC $x10^6/\mu l$	HGB g/dL	HCT %	PLT $x10^3/\mu l$	MCV fL	MCH pg	MCHC %	Retic %	Weight kg	Avg Temp °F	Avg HR /min	Avg Resp /min	serum anti-FHA IU/ml	+/-	serum anti-PTx µg/ml	+/-	serum ADA rel abs	+/-	NPW anti-PTx ng/ml	+/-
1	-33	nd	nd	nd	nd	nd	nd	nd	nd	nd	nd	nd	nd	nd	nd	1.36	nd	nd	nd	19.3	0.6	0.04	0.00	0.12	0.00	nd	nd
7	-27	7.76	4.56	2.78	0.26	0.0	0.1	5.68	16.7	50	594	88	29.4	33.4	0.8	nd	nd	nd	nd	13.0	0.4	144.7	8.7	0.11	0.00	nd	nd
14	-20	8.51	3.40	4.40	0.25	0.1	0.4	5.87	16.7	51.0	516	86.9	28.4	32.7	0.6	nd	nd	nd	nd	16.4	0.7	141.2	0.7	0.11	0.00	nd	nd
21	-13	7.74	2.37	4.73	0.48	0.1	0.0	5.62	15.3	46.9	420	83.5	27.2	32.6	0.9	nd	nd	nd	nd	10.9	1.2	89.6	1.6	0.10	0.00	nd	nd
28	-6	7.73	2.91	4.33	0.35	0.1	0.0	5.52	14.6	44.9	344	81.3	26.4	32.5	1.5	nd	nd	nd	nd	7.9	0.2	63.6	3.9	0.11	0.00	nd	nd
34	0	6.82	3.01	3.55	0.18	0.1	0.0	5.58	14.7	45.6	329	81.7	26.3	32.2	1.1	1.64	98.7	158	51	6.9	0.7	58.2	8.4	0.11	0.00	nd	nd
37	3	8.07	2.82	4.75	0.32	0.2	0.0	5.32	13.9	41.6	319	78.2	26.1	33.4	0.6	1.65	99.3	180	56	8.9	0.2	45.7	7.5	0.11	0.00	4.7	0.2
41	7	6.13	1.72	4.21	0.14	0.0	0.0	5.03	13.1	39.7	327	78.9	26.0	33.0	1.2	1.67	97.9	159	43	7.1	0.2	44.1	1.2	0.11	0.00	nd	nd
44	10	7.96	2.61	5.06	0.20	0.1	0.0	4.90	12.6	39.0	264	79.6	25.7	32.3	2.0	1.74	96.8	170	75	6.5	0.1	74.1	8.6	0.10	0.01	nd	nd
48	14	15.14	7.96	6.62	0.41	0.0	0.1	4.98	12.9	40.3	291	80.9	25.9	32.0	2.1	1.81	96.5	143	48	8.6	0.0	68.6	1.9	0.07	0.00	nd	nd
51	17	8.49	3.23	4.91	0.27	0.1	0.0	4.70	12.0	37.1	322	78.9	25.5	32.3	2.0	1.83	96.7	155	48	15.8	2.1	61.8	7.4	0.10	0.00	nd	nd
55	21	9.89	2.81	6.57	0.41	0.1	0.0	4.90	12.0	37.8	350	77.1	24.5	31.7	1.6	1.99	98.7	211	65	30.5	3.4	59.1	1.4	0.11	0.00	nd	nd
58	24	5.58	1.70	3.63	0.15	0.0	0.1	4.84	12.3	37.8	324	78.1	25.4	32.5	1.9	1.91	97.4	137	42	37.4	3.3	38.4	0.6	0.10	0.00	nd	nd
62	28	5.87	1.64	3.90	0.19	0.1	0.1	5.05	12.8	39.1	317	77.4	25.3	32.7	1.8	1.98	97.6	160	64	54.4	1.7	21.2	1.0	0.10	0.00	nd	nd
65	31	4.60	2.26	2.17	0.16	0.0	0.0	4.90	12.3	38.1	263	77.8	25.1	32.3	2.2	2.02	100.2	151	36	59.5	7.8	19.5	3.4	0.10	0.00	nd	nd
69	35	7.41	2.82	4.15	0.37	0.1	0.0	4.86	12.4	37.0	300	76.1	25.5	33.5	1.6	2.09	100.7	174	38	75.2	1.1	15.6	2.3	0.10	0.00	nd	nd
72	38	4.56	1.41	2.76	0.19	0.1	0.1	4.98	12.8	38.4	332	77.1	25.7	33.3	1.6	2.06	98.4	144	42.7	88.6	0.5	16.7	1.5	0.10	0.00	nd	nd
76	42	5.90	1.52	3.98	0.29	0.1	0.0	4.79	12.2	36.1	358	75.4	25.5	33.8	1.2	2.16	98.6	178	41	93.5	6.9	11.5	0.5	0.10	0.00	nd	nd

Baboon Y3

Age	Challenge Day	WBC Total	WBC Neut	WBC Lymph	WBC Mono	WBC Eosin	WBC Baso	RBC	HGB	HCT	PLT	MCV	MCH	MCHC	Retic	Weight	Avg Temp	Avg HR	Avg Resp	serum anti-FHA		serum anti-PTx		serum ADA		NPW anti-PTx	
days	days	x10^3/µl	x10^3/µl	x10^3/µl	x10^3/µl	x10^3/µl	x10^3/µl	x10^6/µl	g/dL	%	x10^3/µl	fL	pg	%	%	kg	°F	/min	/min	IU/ml	+/-	µg/ml	+/-	rel abs	+/-	ng/ml	+/-
2	-32	nd	nd	nd	nd	nd	nd	nd	nd	nd	nd	nd	nd	nd	nd	0.85	nd	nd	nd	0.5	0.0	0.03	0.00	0.11	0.00	nd	nd
10	-24	5.83	1.96	3.36	0.45	0.1	0.0	5.78	15.3	48.2	421	83.4	26.5	31.7	0.32	nd	nd	nd	nd	0.4	0.0	224.5	10.8	0.11	0.00	nd	nd
17	-17	8.94	3.39	5.02	0.42	0.1	0.0	5.49	14.3	45.3	343	82.5	26.0	31.6	1.2	nd	nd	nd	nd	0.4	0.0	220.4	50.8	0.11	0.01	nd	nd
24	-10	6.80	2.34	4.00	0.39	0.1	0.0	5.18	13.2	42.1	356	81.3	25.5	31.4	3.0	nd	nd	nd	nd	0.4	0.0	117.1	7.2	0.12	0.00	nd	nd
31	-3	6.43	2.15	3.91	0.26	0.1	0.0	5.26	13.2	42.9	385	81.6	25.1	30.8	2.8	nd	nd	nd	nd	0.5	0.0	94.3	6.3	0.13	0.00	nd	nd
34	0	5.54	2.23	2.97	0.18	0.0	0.1	4.66	11.7	36.9	478	79.2	25.1	31.7	3.1	1.15	99.4	184	57	0.6	0.0	105.9	20.5	0.16	0.00	nd	nd
38	4	6.71	1.86	4.14	0.54	0.1	0.1	4.40	11.0	34.6	348	78.6	25.0	31.8	4.8	1.29	99.7	211	66	0.8	0.0	84.0	10.0	0.17	0.00	27.2	5.1
41	7	5.69	2.08	3.23	0.31	0.0	0.0	4.76	11.7	37.1	433	78.1	24.5	31.4	5.6	1.25	98.0	188	69	0.7	0.0	66.2	9.6	0.17	0.00	nd	nd
45	11	6.65	2.33	3.97	0.28	0.0	0.1	4.90	12.1	38.3	407	78.2	24.7	31.6	4.2	1.29	98.3	202	62	0.9	0.0	58.6	2.8	0.15	0.00	nd	nd
48	14	8.14	2.55	5.16	0.37	0.1	0.0	4.85	11.8	38.1	525	78.6	24.3	31.0	3.4	1.38	99.1	216	54	1.6	0.0	67.2	4.5	0.16	0.00	nd	nd
52	18	8.27	2.21	5.57	0.36	0.1	0.1	4.98	12.4	39.3	511	78.9	24.9	31.6	4.5	1.48	99.4	231	66	4.7	0.1	56.0	0.5	0.17	0.00	nd	nd
55	21	6.94	3.44	3.18	0.30	0.0	0.0	5.18	12.6	41.2	449	79.5	24.3	30.6	3.7	1.48	98.5	215	66	12.6	0.2	41.8	0.1	0.19	0.01	nd	nd
59	25	6.90	2.35	4.12	0.39	0.0	0.0	5.03	12.2	39.2	447	77.9	24.3	31.1	2.4	1.60	98.7	214	58	25.4	0.3	36.9	1.2	0.19	0.00	nd	nd
62	28	5.30	1.79	3.24	0.25	0.0	0.0	5.23	12.8	41.2	504	78.8	24.5	31.1	2.9	1.61	99.1	225	72	40.9	1.7	33.7	6.8	0.18	0.01	nd	nd
66	32	5.66	1.39	3.95	0.29	0.0	0.0	5.50	13.5	43.0	429	78.2	24.5	31.4	2.6	1.71	98.7	215	72	59.9	3.2	37.5	2.2	0.19	0.00	nd	nd
69	35	4.59	1.74	2.59	0.25	0.0	0.0	5.31	12.8	41.4	465	78.0	24.1	30.9	2.1	1.70	97.9	169	50	63.4	5.7	15.1	1.5	0.19	0.00	nd	nd
72	38	4.92	2.75	1.97	0.17	0.0	0.0	5.27	12.8	40.8	464	77.4	24.3	31.4	2.7	1.69	98.4	188	42	61.0	2.7	14.5	5.1	0.19	0.00	nd	nd
76	42	6.50	1.98	4.14	0.33	0.0	0.0	5.53	13.3	43.2	421	78.1	24.1	30.8	1.8	nd	98.5	204	40	66.8	1.6	9.5	0.0	0.18	0.00	nd	nd

Baboon Y4

Age	Challenge Day	WBC Total	Neut	Lymph	Mono	Eosin	Baso	RBC	HGB	HCT	PLT	MCV	MCH	MCHC	Retic	Weight	Avg Temp	Avg HR	Avg Resp	serum anti-FHA		serum anti-PTx		serum ADA		NPW anti-PTx	
days	days	x10^3/µl	x10^3/µl	x10^3/µl	x10^3/µl	x10^3/µl	x10^3/µl	x10^6/µl	g/dL	%	x10^3/µl	fL	pg	%	%	kg	°F	/min	/min	IU/ml	+/-	µg/ml	+/-	rel abs	+/-	ng/ml	+/-
2	-29	9.12	7.00	1.37	0.73	0.0	0.0	5.49	16.6	51.3	419	93.4	30.2	32.4	2.2	nd	98.0	131	36	0.5	0.0	0.1	0.0	0.12	0.00	nd	nd
7	-24	5.66	1.65	3.32	0.61	0.1	0.0	5.39	15.9	48.2	484	89.4	29.5	33.0	0.4	nd	nd	nd	nd	0.3	0.0	272.7	28.4	0.11	0.00	nd	nd
14	-17	8.28	2.70	5.01	0.47	0.1	0.0	5.05	14.4	44.5	574	88.1	28.5	32.4	1.4	nd	nd	nd	nd	0.4	0.0	215.4	42.7	0.11	0.00	nd	nd
21	-10	7.97	1.39	5.81	0.68	0.1	0.0	4.94	13.7	41.9	100	84.8	27.7	32.7	1.3	nd	nd	nd	nd	0.6	0.1	130.6	19.6	0.14	0.00	nd	nd
28	-3	8.6	1.32	6.75	0.45	0.1	0.0	4.89	13.3	41.1	400	84.0	27.2	32.4	1.1	nd	nd	nd	nd	1.1	0.1	95.0	6.2	0.15	0.00	nd	nd
31	0	9.61	5.62	3.46	0.39	0.0	0.1	4.24	11.4	34.3	430	80.9	26.9	33.2	1.3	1.19	98.0	154	63	0.3	0.0	105.5	9.0	0.16	0.00	nd	nd
35	4	8.05	2.04	5.21	0.69	0.0	0.1	3.99	10.7	32.2	433	80.7	26.8	33.2	2.2	1.28	99.7	185	52	0.5	0.0	76.8	7.5	0.17	0.01	2.4	0.2
38	7	8.5	3.44	4.57	0.43	0.0	0.0	4.01	10.5	31.7	407	79.1	26.2	33.1	3.4	1.28	97.3	154	69	0.6	0.0	57.8	5.3	0.15	0.00	nd	nd
42	11	8.25	1.73	6.17	0.32	0.0	0.0	4.17	10.8	33.3	422	79.9	25.9	32.4	3.3	1.35	97.5	142	64	0.4	0.0	49.0	1.8	0.18	0.00	nd	nd
45	14	10.53	2.57	7.42	0.48	0.0	0.0	4.18	10.8	33.6	443	80.4	25.8	32.1	3.5	1.41	98.9	193	90	0.9	0.1	71.2	11.8	0.21	0.00	nd	nd
49	18	12.16	4.10	7.54	0.40	0.0	0.1	3.97	10.4	31.3	473	78.8	26.2	33.2	2.9	1.48	99.6	196	60	1.6	0.7	49.5	1.6	0.22	0.03	nd	nd
52	21	8.78	2.63	5.76	0.35	0.0	0.0	4.19	10.7	33.3	469	79.5	25.5	32.1	2.7	1.55	98.6	183	70	3.5	0.0	56.7	6.9	0.16	0.00	nd	nd
56	25	6.42	1.69	4.43	0.27	0.0	0.0	4.41	11.2	34.8	498	78.9	25.4	32.2	2.7	1.57	98.3	187	56	6.8	0.1	91.0	7.8	0.15	0.02	nd	nd
59	28	5.59	1.37	3.88	0.31	0.0	0.0	4.39	11.3	34.6	509	78.8	25.7	32.7	2.2	1.59	97.7	183	54	20.2	0.1	66.6	1.7	0.16	0.01	nd	nd
63	32	8.03	2.04	5.47	0.45	0.0	0.0	4.58	11.7	35.3	436	77.1	25.5	33.1	1.8	1.74	98.4	158	64	32.1	1.9	57.1	3.2	0.16	0.01	nd	nd
66	35	5.4	1.46	3.67	0.26	0.0	0.0	4.46	11.4	35.0	510	78.5	25.6	32.6	2.2	1.68	97.9	175	42	41.9	1.2	48.2	2.0	0.16	0.00	nd	nd
69	38	5.11	1.33	3.56	0.19	0.0	0.0	4.45	11.2	34.3	500	77.1	25.2	32.7	2.3	1.70	97.9	156	42	41.0	1.0	44.5	1.5	0.15	0.01	nd	nd
73	42	6.09	1.27	4.57	0.21	0.0	0.0	4.47	11.3	34.8	510	77.9	25.3	32.5	1.4	nd	98.1	155	38	54.5	3.8	45.2	0.4	0.14	0.00	nd	nd

Baboon Y5

		WBC																		serum		serum		serum		NPW	
Age	Challenge Day	Total	Neut	Lymph	Mono	Eosin	Baso	RBC	HGB	HCT	PLT	MCV	MCH	MCHC	Retic	Weight	Avg Temp	Avg HR	Avg Resp	anti-FHA		anti-PTx		ADA		anti-PTx	
days	days	$x10^3/\mu l$	$x10^3/\mu l$	$x10^3/\mu l$	$x10^3/\mu l$	$x10^3/\mu l$	$x10^3/\mu l$	$x10^6/\mu l$	g/dL	%	$x10^3/\mu l$	fL	pg	%	%	kg	°F	/min	/min	IU/ml	+/-	µg/ml	+/-	rel abs	+/-	ng/ml	+/-
1	-34	13.12	10.69	1.01	1.40	0.0	0.0	4.55	16.2	45.6	197	100.0	35.6	35.5	9.0	1.22	96.7	160	56	0.5	0.0	0.2	0.0	0.13	0.00	nd	nd
7	-28	nd	nd	nd	nd	nd	nd	nd	nd	nd	nd	nd	nd	nd	nd	nd	nd	nd	nd	0.4	0.0	296.0	7.1	0.12	0.00	nd	nd
13	-22	nd	nd	nd	nd	nd	nd	nd	nd	nd	nd	nd	nd	nd	nd	nd	nd	nd	nd	0.5	0.0	253.9	33.6	0.14	0.00	nd	nd
21	-14	nd	nd	nd	nd	nd	nd	nd	nd	nd	nd	nd	nd	nd	nd	nd	nd	nd	nd	0.4	0.0	242.5	8.3	0.20	0.01	nd	nd
28	-7	nd	nd	nd	nd	nd	nd	nd	nd	nd	nd	nd	nd	nd	nd	nd	nd	nd	nd	0.3	0.0	146.8	12.5	0.31	0.03	nd	nd
35	0	7.05	3.15	3.82	0.01	0.0	0.0	5.01	13.1	39.8	382	79.4	26.1	32.9	4.1	1.59	99.4	157	44	0.2	0.1	61.6	4.8	0.48	0.03	nd	nd
38	3	11.88	3.12	8.24	0.46	0.0	0.0	5.24	13.5	41.6	348	79.4	25.8	32.5	3.2	1.63	98.8	194	64	0.7	0.0	26.9	0.2	0.49	0.00	1.3	0.0
42	7	26.61	5.08	20.46	0.82	0.2	0.1	5.15	13.2	41.1	391	79.8	25.6	32.1	3.7	1.69	98.7	176	62	0.7	0.0	1.0	0.0	0.52	0.03	nd	nd
45	10	33.28	7.55	24.86	0.63	0.2	0.0	4.72	12.0	37.8	383	80.1	25.4	31.7	3.9	1.78	98.4	187	72	1.1	0.0	0.2	0.0	0.51	0.02	nd	nd
49	14	38.39	9.75	27.10	1.27	0.2	0.1	4.91	12.5	39.3	370	80.0	25.5	31.8	3.4	1.80	98.9	165	52	13.9	0.7	0.7	0.1	0.51	0.02	nd	nd
52	17	26.51	8.75	16.57	1.11	0.1	0.0	5.05	12.8	40.5	396	80.2	25.3	31.6	3.6	1.88	98.5	190	58	38.4	1.2	10.5	0.8	0.59	0.01	nd	nd
56	21	13.04	4.58	7.84	0.55	0.0	0.0	4.77	11.9	37.3	447	78.2	24.9	31.9	2.9	1.86	98.6	180	43	108.6	3.6	62.6	2.2	0.59	0.00	nd	nd
59	24	13.92	7.14	6.17	0.58	0.0	0.0	4.74	11.9	36.7	498	77.4	25.1	32.4	2.1	1.90	98.3	172	38	153.9	0.6	62.4	5.4	0.58	0.00	nd	nd
63	28	8.35	3.83	4.16	0.30	0.0	0.0	4.95	12.4	38.3	496	77.4	25.1	32.4	3.5	1.96	98.7	170	44	180.9	3.3	55.8	4.1	0.57	0.01	nd	nd
66	31	12.74	9.53	2.79	0.39	0.0	0.0	4.74	11.8	37.2	453	78.5	24.9	31.7	2.9	2.00	98.7	177	44	151.2	13.2	48.5	7.6	0.56	0.01	nd	nd
70	35	7.66	5.38	2.01	0.25	0.0	0.0	4.91	12.2	38.3	387	78.0	24.8	31.9	2.8	2.04	98.8	163	34	138.6	20.2	42.4	2.5	0.54	0.03	nd	nd
73	38	6.87	2.96	3.66	0.23	0.0	0.0	4.99	12.2	38.8	383	77.8	24.4	31.4	2.6	nd	99.1	139	36	207.3	11.1	34.5	2.6	0.55	0.03	nd	nd
77	42	9.74	8.14	1.43	0.17	0.0	0.0	4.92	12.1	38.3	356	77.8	24.6	31.6	2.7	nd	98.7	173	34	195.6	8.8	26.1	0.5	0.55	0.01	nd	nd

Baboon Y6

Age<br>days	Challenge Day<br>days	WBC Total<br>x10³/µl	Neut<br>x10³/µl	Lymph<br>x10³/µl	Mono<br>x10³/µl	Eosin<br>x10³/µl	Baso<br>x10³/µl	RBC<br>x10⁶/µl	HGB<br>g/dL	HCT<br>%	PLT<br>x10³/µl	MCV<br>fL	MCH<br>pg	MCHC<br>%	Retic<br>%	Weight<br>kg	Avg Temp<br>°F	Avg HR<br>/min	Avg Resp<br>/min	serum anti-FHA<br>IU/ml	+/-	serum anti-PTx<br>µg/ml	+/-	serum ADA<br>rel abs	+/-	NPW anti-PTx<br>ng/ml	+/-
1	-28	8.98	7.35	1.07	0.55	0.0	0.0	4.44	13.9	40.7	370	91.7	31.3	34.2	2.8	0.76	98.0	144	52	1.3	0.1	0.6	0.0	0.13	0.00	nd	nd
7	-22	nd	nd	nd	nd	nd	nd	nd	nd	nd	nd	nd	nd	nd	nd	nd	nd	nd	nd	1.0	0.0	342.1	5.8	0.13	0.00	nd	nd
15	-14	nd	nd	nd	nd	nd	nd	nd	nd	nd	nd	nd	nd	nd	nd	nd	nd	nd	nd	0.7	0.0	208.9	4.1	0.13	0.00	nd	nd
22	-7	nd	nd	nd	nd	nd	nd	nd	nd	nd	nd	nd	nd	nd	nd	nd	nd	nd	nd	0.5	0.0	162.4	9.3	0.10	0.02	nd	nd
29	0	5.43	1.94	3.14	0.30	0.0	0.0	4.36	11.60	34.60	494.00	79.40	26.60	33.50	2.07	1.02	99.80	150.00	42.00	0.6	0.0	115.5	13.8	0.15	0.00	nd	nd
32	3	7.95	3.39	3.48	0.97	0.1	0.0	4.36	11.1	34.1	397	78.2	25.5	32.6	1.4	1.05	98.7	172	56	3.6	0.2	108.6	13.4	0.15	0.01	0.9	0.1
36	7	8.96	3.21	4.92	0.76	0.0	0.0	4.39	11.1	34.7	443	79.0	25.3	32.0	3.9	1.10	99.0	191	60	3.3	0.0	85.5	4.7	0.16	0.00	nd	nd
39	10	13.75	6.23	6.75	0.70	0.1	0.0	4.73	11.7	37.4	466	79.1	24.7	31.3	3.8	1.10	98.9	155	38	3.4	0.1	83.5	2.8	0.16	0.00	nd	nd
43	14	13.53	3.99	8.70	0.77	0.1	0.0	4.55	11.4	36.0	442	79.1	25.1	31.7	3.9	1.19	99.2	159	38	3.5	0.2	75.2	12.3	0.14	0.00	nd	nd
46	17	10.75	3.03	7.18	0.48	0.1	0.0	4.78	11.8	37.7	465	78.9	24.7	31.3	3.4	1.22	99.1	172	42	4.7	0.0	59.4	6.5	0.14	0.00	nd	nd
50	21	9.86	5.24	4.15	0.40	0.0	0.0	4.96	12.2	38.2	487	77.0	24.6	31.9	2.2	1.24	98.7	158	42	13.8	0.4	46.1	0.8	0.14	0.01	nd	nd
53	24	7.73	2.72	4.54	0.43	0.0	0.0	5.09	12.4	39.0	421	76.6	24.4	31.8	1.8	1.28	98.3	140	40	17.3	1.4	51.3	8.3	0.13	0.01	nd	nd
57	28	6.15	2.51	3.28	0.33	0.0	0.0	4.91	11.9	37.2	428	75.8	24.2	32.0	2.1	1.30	98.5	138	40	22.2	1.3	50.9	6.6	0.14	0.01	nd	nd
60	31	7.46	3.45	3.53	0.46	0.0	0.0	4.97	12.1	37.8	420	76.1	24.3	32.0	2.1	1.33	98.9	166	40	33.3	2.2	40.1	1.9	0.13	0.00	nd	nd
64	35	7.13	3.44	3.23	0.44	0.0	0.0	5.32	12.7	40.0	414	75.2	23.9	31.8	1.9	1.36	98.0	167	54	40.6	2.2	30.0	3.9	0.13	0.00	nd	nd
67	38	6.3	2.15	3.74	0.36	0.0	0.0	4.95	11.9	37.3	428	75.4	24.0	31.9	1.7	nd	99.0	187	46	46.5	0.1	23.2	1.6	0.15	0.03	nd	nd
71	42	7.21	2.47	4.10	0.59	0.0	0.0	4.89	11.8	37.0	399	75.7	24.1	31.9	2.4	nd	98.4	154	38	40.3	2.1	25.1	5.7	0.13	0.00	nd	nd

Baboon Y7

Age days	Challenge Day days	WBC Total $x10^3$/µl	WBC Neut $x10^3$/µl	WBC Lymph $x10^3$/µl	WBC Mono $x10^3$/µl	WBC Eosin $x10^3$/µl	WBC Baso $x10^3$/µl	RBC $x10^6$/µl	HGB g/dL	HCT %	PLT $x10^3$/µl	MCV fL	MCH pg	MCHC %	Retic %	Weight kg	Avg Temp °F	Avg HR /min	Avg Resp /min	serum anti-FHA IU/ml	+/-	serum anti-PTx µg/ml	+/-	serum ADA rel abs	+/-	NPW anti-PTx ng/ml	+/-
1	-32	9.77	8.56	0.62	0.58	0.0	0.0	3.38	10.7	34.0	263	101.0	31.7	31.5	7.0	0.98	98.3	147	68	0.5	0.0	0.1	0.0	0.12	0.00	nd	nd
7	-26	7.08	3.83	2.81	0.36	0.06	0.02	3.66	10.8	33.6	820	91.8	29.5	32.1	2.4	nd	nd	nd	nd	0.6	0.1	220.8	46.1	0.11	0.01	nd	nd
14	-19	6.16	3.44	2.39	0.28	0.02	0.03	4.11	11.5	36.1	620	87.8	28.0	31.9	2.7	nd	nd	nd	nd	0.5	0.0	368.4	34.5	0.12	0.00	nd	nd
21	-12	6.59	3.19	3.03	0.31	0.04	0.02	4.46	12.1	38.0	345	85.2	27.1	31.8	3.6	nd	nd	nd	nd	0.5	0.1	241.1	40.4	0.14	0.01	nd	nd
28	-5	nd	nd	nd	nd	nd	nd	nd	nd	nd	nd	nd	nd	nd	nd	nd	nd	nd	nd	0.8	0.1	180.0	12.7	0.16	0.00	nd	nd
33	0	4.14	2.81	1.22	0.10	0.0	0.0	4.68	12.2	37.1	427	79.3	26.1	32.9	1.8	1.20	99.9	165	38	1.1	0.1	142.6	3.6	0.18	0.01	nd	nd
36	3	5.56	2.32	2.82	0.38	0.0	0.0	4.61	11.9	36.6	358	79.4	25.8	32.5	1.2	1.26	98.3	167	100	1.2	0.3	102.2	7.4	0.21	0.01	23.5	3.1
40	7	9.46	5.57	3.15	0.69	0.0	0.0	4.66	11.8	36.7	366	78.8	25.3	32.2	2.5	1.29	98.4	145	66	1.4	0.1	151.1	9.9	0.23	0.00	nd	nd
43	10	14.22	7.69	5.96	0.51	0.1	0.0	4.74	12.0	37.5	386	79.1	25.3	32.0	2.5	1.35	99.3	213	92	1.6	0.1	149.8	28.9	0.18	0.08	nd	nd
47	14	9.37	3.62	5.44	0.28	0.0	0.0	4.79	12.1	38.5	342	80.4	25.3	31.4	2.2	1.41	98.4	169	74	2.3	0.1	120.9	27.1	0.20	0.06	nd	nd
50	17	10.35	4.77	5.29	0.27	0.0	0.0	4.81	12.3	38.6	398	80.2	25.6	31.9	2.1	1.46	98.5	186	78	4.9	0.1	88.7	3.7	0.23	0.01	nd	nd
54	21	7.05	3.15	3.58	0.29	0.0	0.0	5.08	12.7	39.8	383	78.3	25.0	31.9	1.5	1.49	98.7	189	80	26.5	0.2	83.4	6.6	0.25	0.00	nd	nd
57	24	8.05	5.64	2.00	0.40	0.0	0.0	5.14	12.7	40.5	378	78.8	24.7	31.4	1.4	1.50	97.8	184	66	108.5	7.7	93.8	5.0	0.26	0.00	nd	nd
61	28	7	3.46	3.12	0.35	0.1	0.0	5.20	13.0	40.0	369	76.9	25.0	32.5	1.4	1.51	98.2	153	72	221.7	5.9	86.6	30.6	0.28	0.00	nd	nd
64	31	6.5	2.72	3.44	0.28	0.1	0.0	5.44	13.6	42.3	414	77.8	25.0	32.2	1.1	1.53	99.1	155	76	261.5	8.7	72.8	15.5	0.30	0.01	nd	nd
68	35	9.44	6.77	2.41	0.23	0.0	0.0	5.31	13.2	40.9	330	77.0	24.9	32.3	0.9	1.56	99.0	177	74	302.7	6.8	56.6	8.8	0.39	0.02	nd	nd
71	38	6.03	3.50	2.29	0.21	0.0	0.0	4.92	12.1	37.8	344	76.8	24.6	32.0	1.0	1.59	98.7	180	54	255.2	9.6	41.5	6.2	0.40	0.02	nd	nd
75	42	5.89	3.18	2.45	0.23	0.0	0.0	5.02	12.4	38.8	342	77.3	24.7	32.0	1.8	1.61	98.7	176	66	214.5	11.3	71.1	11.8	0.42	0.01	nd	nd
